# Virtual Reality Distraction for Reducing Pain and Anxiety During Percutaneous Cardiovascular Interventions: A Systematic Review and Meta-Analysis with Trial Sequential Analysis

**DOI:** 10.3390/medicina61060957

**Published:** 2025-05-22

**Authors:** Ebraheem Albazee, Abdullhadi Alrajehi, Fahad M. Alsahli, Abdillatef Alqemlas, Ahmad Aldhaen, Abdullah Alkandari, Hamad Alkandari, Waleed Alkanderi

**Affiliations:** 1 Otorhinolaryngology-Head and Neck Surgery, Kuwait Institute for Medical Specializations (KIMS), Kuwait City 13018, Kuwait; 2 Department of Otolaryngology-Head and Neck Surgery, Al-Jahra Hospital, Al Jahra 03200, Kuwait; 3 Faculty of Medicine, University of Jordan, Amman 11942, Jordan; hadialrajhi@me.com (A.A.); fahadalsahli98@gmail.com (F.M.A.); abdullatifalqamlas@gmail.com (A.A.); ahmad.aldhaen03@gmail.com (A.A.); abo0od.02@live.com (A.A.); hsk_alk@hotmail.com (H.A.); 4 Kuwait Institute for Medical Specializations (KIMS), Kuwait City 13018, Kuwait; waleedalkanderiii@gmail.com

**Keywords:** analgesia, coronary angiography, PCI, sedation, TAVR

## Abstract

*Background and Objectives:* Percutaneous cardiovascular interventions (PCIs) have become a cornerstone in the management of cardiovascular diseases. However, patients often experience significant anxiety and pain during these procedures, which can negatively impact their overall experience and clinical outcomes. Virtual reality (VR) is an emerging non-pharmacological intervention designed to alleviate procedural anxiety and pain through immersive distraction techniques. *Materials and Methods:* We conducted a systematic review and meta-analysis of randomized controlled trials (RCTs) identified from PubMed, CENTRAL, Scopus, Google Scholar, and Web of Science up to November 2024. Primary outcomes were peri-procedural anxiety and pain; secondary outcomes included vital signs, procedure duration, and safety (e.g., delirium). Continuous data were pooled using a random-effect model and reported as standardized mean differences (SMDs) with 95% confidence intervals (CIs) in Stata MP v.17. Certainty of evidence was assessed using the GRADE approach. *Results:* Ten RCTs involving 890 patients were included. VR distraction significantly reduced peri-procedural anxiety (SMD: –0.70; 95% CI: –1.15 to –0.26; *p* < 0.001). However, no significant differences were observed between groups for peri-procedural pain (SMD: –0.64; 95% CI: –1.45 to 0.16; *p* = 0.12), systolic blood pressure (SMD: –0.31; 95% CI: –1.23 to 0.61; *p* = 0.50), diastolic blood pressure (SMD: –0.25; 95% CI: –1.07 to 0.56; *p* = 0.54), heart rate (SMD: –0.44; 95% CI: –0.93 to 0.05; *p* = 0.08), respiratory rate (SMD: –0.93; 95% CI: –2.18 to 0.31; *p* = 0.14), or procedure duration (SMD: 0.07; 95% CI: –1.14 to 0.28; *p* = 0.49). *Conclusions:* VR significantly ameliorated peri-procedure anxiety in patients undergoing PCIs; however, it had no effect on peri-procedure pain or vital signs. This is based on uncertain evidence from heterogeneous studies, warranting further confirmation through large-scale RCTs.

## 1. Introduction

The prevalence of pain and anxiety is substantial among patients undergoing percutaneous cardiac interventions (PCIs) [1]. Anxiety is a prevalent concern, impacting 40% to 80% of patients throughout all stages of PCIs (pre-, peri-, and post-procedure) [1,2,3,4]. Additionally, pain is a frequent side effect for patients undergoing PCIs, affecting 40% to 75% of patients during and following the procedure [1,5,6,7]. The experience of both pain and anxiety triggers stress hormone release, resulting in a subsequent elevation of pro-inflammatory cytokine levels [8,9,10,11]. Consequently, multiple complications (myocardial injury, renal injury, cerebrovascular events, impaired wound healing, arrhythmias, depression, and delirium) can develop as a result of these physiological changes [1,12,13].

Conventional interventions to manage PCI-associated pain and anxiety include pharmacological analgesia, conscious sedation, and non-pharmacological measures such as patient education [1]. Nevertheless, these measures can have limited efficacy in some cases and an increased risk of side effects, including tolerance or dependence [14]. In particular, conscious sedation is commonly employed during coronary angiography (CAG) to mitigate patient anxiety and pain, also reducing the incidence of arterial spasms [15,16]. Nonetheless, potential adverse effects include hypoxemia, extended recovery, cognitive impairment, amnesia, and the possible requirement of an antagonist (flumazenil) [17]. Accordingly, a growing demand for non-pharmacological interventions has emerged as a necessary approach to mitigate adverse effects associated with conventional interventions [1].

Virtual reality (VR), a recently introduced strategy, aims to divert patients’ attention away from painful medical procedures, thereby reducing their processing of nociceptive stimuli [8], thus serving as a supplemental therapy for pain and anxiety management. VR is delivered through a high-definition screen integrated into a head-mounted display, enabling the user to become fully immersed in a three-dimensional world; some versions even provide hypnotic suggestions through a recorded voice that guides the patient [18]. The efficacy of VR in reducing pain and anxiety has already been demonstrated in several clinical interventions [8,19]. Integrating immersive visual and auditory stimuli within VR generates an engaging setting, effectively redirecting patients’ attention from procedural anxiety. VR can be administered in pre-, peri-, and post-procedural phases of PCIs [1].

Recently, several randomized controlled trials (RCTs) have reported conflicting results regarding the effect of VR on pain and anxiety associated with PCIs, including CAG, transcatheter aortic valve replacement (TAVR), and implantable cardiac defibrillator (ICD) procedures [18,20,21,22,23,24,25,26,27,28]. Hence, this systematic review and meta-analysis aim to provide a comprehensive evaluation of the impact of virtual reality on pain and anxiety in patients undergoing percutaneous cardiovascular interventions.

## 2. Materials and Methods

### 2.1. Protocol Registration

Before the review process, this review was registered on the International Prospective Register of Systematic Reviews (PROSPERO) with the CRD420251036908. This systematic review and meta-analysis were conducted in accordance with the Preferred Reporting Items for Systematic Reviews and Meta-Analyses (PRISMA) statement [29] and the Cochrane Handbook for Systematic Reviews and Meta-Analyses [30].

### 2.2. Data Sources and Search Strategy

On 16th November 2024, an electronic search was conducted on the following databases: Web of Science (WOS), PubMed, Scopus, Google Scholar, and CENTRAL. The search strategy incorporated the following entry terms “(“virtual reality” OR “smart glass*” OR “immersive” OR “non-immersive” OR “head-mounted display” OR “augmented reality” OR “mixed reality” OR “virtual therapy” OR “virtual environment” OR “virtual treatment” OR “visual distract*” OR “audiovisual distract*” OR “photic stimulation” OR “motion picture*” OR “watch* video*”) AND (“interventional cardiology” OR “cardiac intervention” OR “percutaneous cardiac procedure*” OR “percutaneous coronary intervention” OR “PCI” OR “cardiac catheter*” OR “coronary angiograph*” OR “cardiac device implant*” OR “pacemaker implant*” OR “cardiac ablation” OR “TAVR” OR “TAVI” OR “transcatheter aortic valve replacement” OR “transcatheter aortic valve implantation” OR “endovascular procedure*”)”. Our search was unconstrained, except for Scopus, where we limited the search scope to titles and abstracts. Each database’s entry terms and search results are demonstrated in Table S1. To ensure a complete review and avoid the exclusion of any eligible records, a thorough manual search of the trial list references was undertaken.

### 2.3. Eligibility Criteria

RCTs were included if they met the following PICO criteria: the population consisted of adult patients (>18 years) undergoing percutaneous cardiovascular interventions, including diagnostic or therapeutic CAG, PCI, TAVR, catheter ablation, ICD, or pacemaker implantation. The intervention involved the use of VR distraction techniques applied before or during the procedure, and the control group received standard care without VR. The primary outcomes were peri-procedural anxiety and pain, assessed using any validated instrument. Secondary outcomes included vital signs (systolic blood pressure [SBP], diastolic blood pressure [DBP], heart rate [HR], and respiratory rate [RR]), procedure duration, and safety outcomes, including the incidence of delirium. Studies were excluded if they employed VR for patient education rather than distraction, were quasi-randomized, observational, or in vitro studies, or were published as conference abstracts, proceedings, or reviews.

### 2.4. Study Selection

The study selection process was conducted using the Covidence online platform. Two independent reviewers performed a two-stage screening process. After removing duplicate records, all unique citations were first screened by title and abstract. Records deemed potentially eligible then underwent full-text review. Any discrepancies between reviewers were resolved through discussion to reach consensus.

### 2.5. Data Extraction

To design an Excel extraction form, the full texts of all relevant publications were first obtained; this allowed for a pilot extraction. The form incorporated three sections: included trials’ summary characteristics (study ID, country, study design, number of centres, total patients, VR protocols, procedure type, sedation, distraction timing, and outcomes assessment tools); included participants’ baseline characteristics (age, gender, body mass index (BMI), and comorbidities); and the outcome: primary outcomes (peri-procedural anxiety and pain), and secondary outcomes: vital signs (SBP, DBP, HR, and RR), procedure duration, and safety.

Data extraction was independently performed by two reviewers, with any discrepancies subsequently resolved through discussion and consensus with a senior author. Event and total formats were used for extracting dichotomous outcome variables, while mean and standard deviation were used for continuous outcome variables. We utilized the formulas provided by Wan et al. [31] to convert the data from median and interquartile range or range to mean and standard deviation.

### 2.6. Risk of Bias and Certainty of Evidence

We used the revised Cochrane Collaboration tool for RCTs (ROB 2) to assess the risk of bias in included studies [32]. Two reviewers independently assessed each study, evaluating its selection criteria, performance quality, reporting methods, attrition rates, and overall biases; disagreements were resolved through a consensus-building process. Also, the certainty of evidence was assessed using the Grading of Recommendations Assessment, Development, and Evaluation (GRADE) framework, which considered factors like inconsistency, imprecision, indirectness, publication bias, and risk of bias [33,34]. Each factor was individually evaluated, and the decisions were duly justified and documented. Any inconsistencies were resolved through discussion.

### 2.7. Statistical Analysis

The statistical analysis was performed using Stata MP v. 17 by Stata Corp. We utilized the risk ratio (RR) to combine dichotomous outcomes and the mean difference (MD) to combine continuous outcomes, along with a 95% confidence interval (CI). We utilized the fixed-effect model unless there was significant heterogeneity, in which case we employed the random-effect model. An assessment of the statistical heterogeneity among the included studies was conducted using the chi-squared test and the I-squared statistic (I^2^); we defined statistical significance using a threshold of *p* < 0.1 for the chi-square test alongside an I^2^ value of 50% or higher to represent significant heterogeneity. A sensitivity analysis was conducted using the leave-one-out model to account for significant heterogeneity. By excluding each study individually, the potential impact on the overall effect estimate was observed, ensuring that no single study had disproportionate influence. The Galbraith plot was also utilized to identify any variation among the studies.

We also conducted a subgroup analysis based on the type of procedure and timing of the VR application. Publication bias was not investigated, as all assessed outcomes had less than 10 RCTs [35]. Finally, trial sequential analysis (TSA) was conducted to evaluate the robustness and conclusiveness of the meta-analytic results. To determine the sufficiency and robustness of the available evidence, the TSA considers the information’s size and the cumulative z-curve. Boundary controls were established to manage the risks associated with Type I and Type II errors. TSA was conducted using the trial sequential analysis software [36].

## 3. Results

### 3.1. Search Results and Study Selection

**Figure 1:**
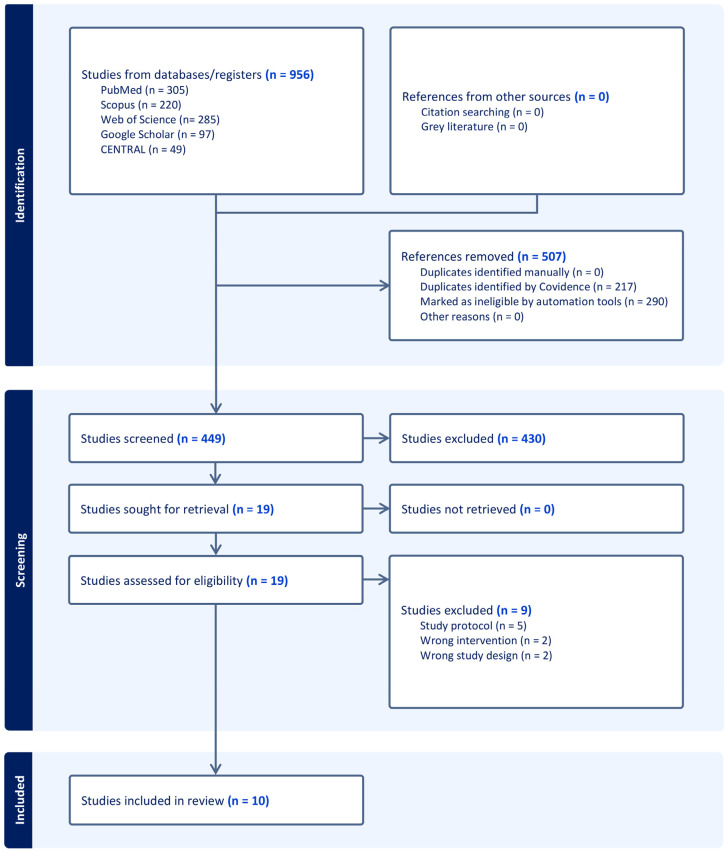
PRISMA (Preferred Reporting Items for Systematic Reviews and Meta-Analyses) flow diagram illustrating the study selection process.

Following a literature search, 956 studies were identified and screened based on title and abstract. Following title and abstract screening, 507 irrelevant records and 430 studies failing to meet the inclusion criteria were excluded, leaving 19 full-text articles for further analysis. Nine studies were excluded, leaving ten to be assessed in qualitative and quantitative analysis [18,20,21,22,23,24,25,26,27,28], as shown in Figure 1. The details of the excluded records during full-text screening are outlined in Table S2.

### 3.2. Characteristics of Included Studies

Ten RCTs and 890 patients were included [18,20,21,22,23,24,25,26,27,28]. Five trials included patients undergoing CAG [20,21,23,25,28], three included patients undergoing TAVR [22,24,26], one included patients undergoing ICD [18], and another included patients undergoing CAG, coronary angioplasty, or peripheral angioplasty [27]. Four trials provided additional sedation [18,24,26,27], and one trial used no sedation [22], with no information about sedation in other trials [20,21,23,25,28]. Further information about trial design is highlighted in Table 1. The VR group had 445 patients, while the control group also had 445 patients. Additional information about the included patients is highlighted in Table 2.

### 3.3. Risk of Bias and Certainty of Evidence

**Figure 2:**
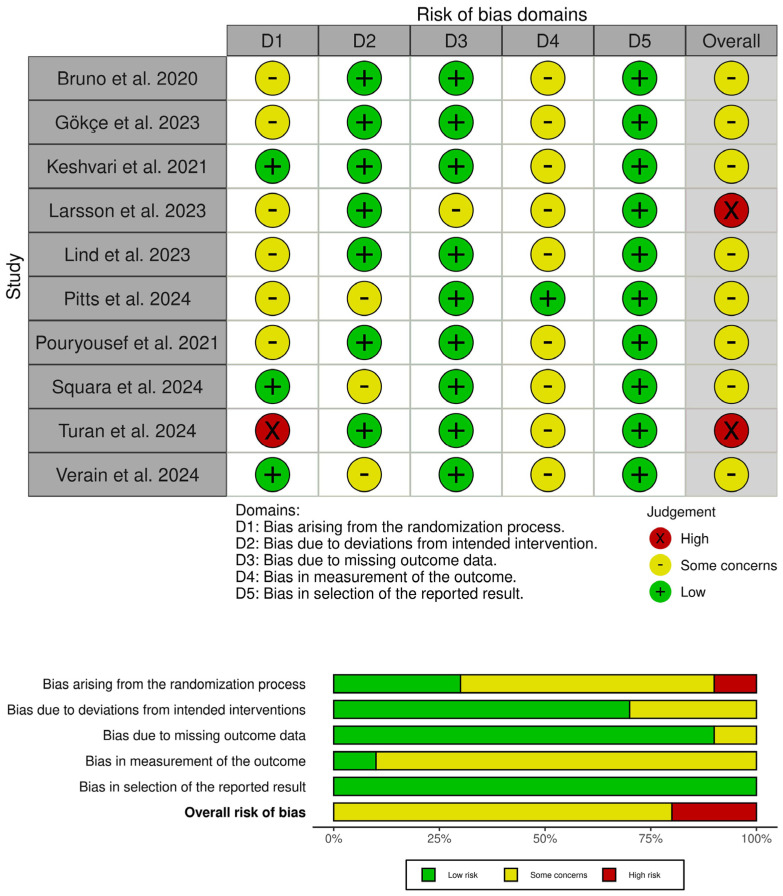
Risk of bias assessment of the included trials. The upper panel presents a study-level summary of bias judgments across individual domains (green = low risk, yellow = some concerns, red = high risk). The lower panel provides an aggregated overview of risk levels across all domains for the included trials [18,20,21,22,23,24,25,26,27,28].

Eight studies had some concerns about overall bias [18,20,22,24,25,26,27,28], with two trials having a high risk of overall bias [21,23], as shown in Figure 2. Regarding selection bias, six trials had some concerns due to the lack of information about the randomization process [20,22,23,24,26,28], with Turan et al. indicating that they used the patient’s record number for randomization; even numbers were randomized to intervention and odd numbers to control [21]. Regarding performance bias, three trials showed some concerns as they used adjuvant analgesia, which may have differed from patient to patient, given the interventions’ open-label nature [18,24,27]. Larsson et al. expressed some concerns about attrition bias due to a significant loss of follow-up in the VR group (14 patients) without a clear rationale [23]. Finally, nine trials showed some concerns of detection bias due to the open-label assessment of subjective outcomes [18,20,21,22,23,25,26,27,28]. Furthermore, details on the certainty of evidence assessment are shown in Table 3.

### 3.4. Primary Outcomes: Peri-Procedural Anxiety and Pain

**Figure 3:**
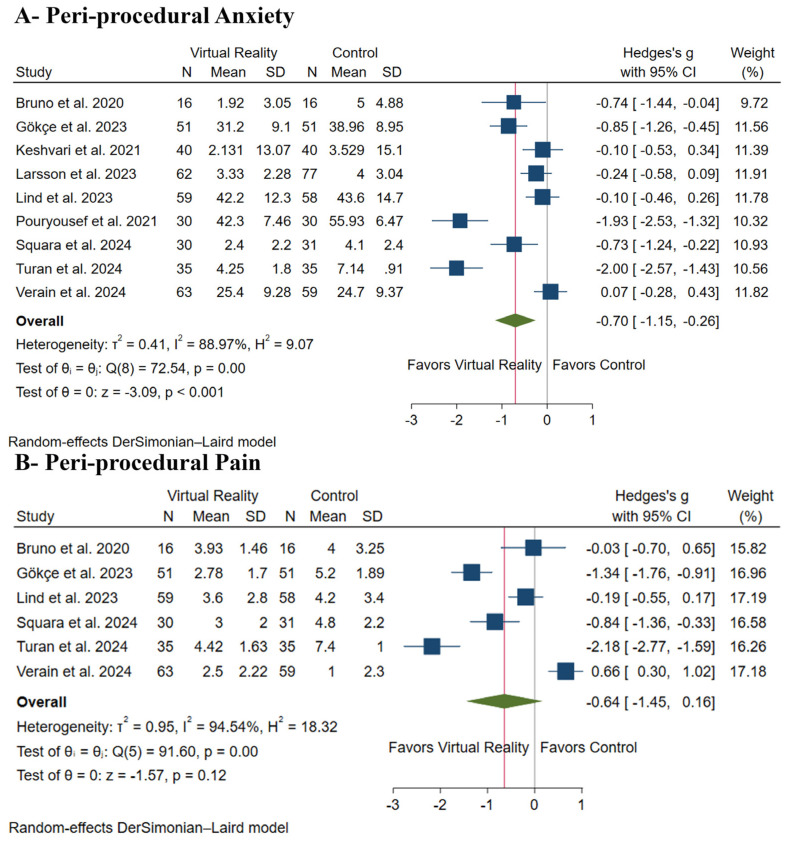
Forest plots of the primary outcomes ((**A**)—peri-procedure anxiety; (**B**)—peri-procedure pain), CI: confidence interval [18,20,21,22,23,25,26,27,28].

VR distraction significantly decreased peri-procedural anxiety (SMD: −0.70, with 95% CI [−1.15, −0.26], *p* < 0.001), as shown in Figure 3A; however, there was no difference between both groups regarding peri-procedural pain (SMD: −0.64, with 95% CI [−1.45, 0.16], *p* = 0.12), as shown in Figure 3B. Pooled studies were heterogeneous in peri-procedural anxiety (I2 = 89%, *p* < 0.001) and peri-procedural pain (I2 = 95%, *p* < 0.001). Leave-one-out sensitivity analysis showed consistent results in each scenario in peri-procedural anxiety, as shown in Figure S1; however, VR distraction significantly decreased peri-procedural pain after excluding Verain et al. (SMD: −0.92, with 95% CI [−1.64, 0.19], *p* = 0.01), as shown in Figure S2.

**Figure 4:**
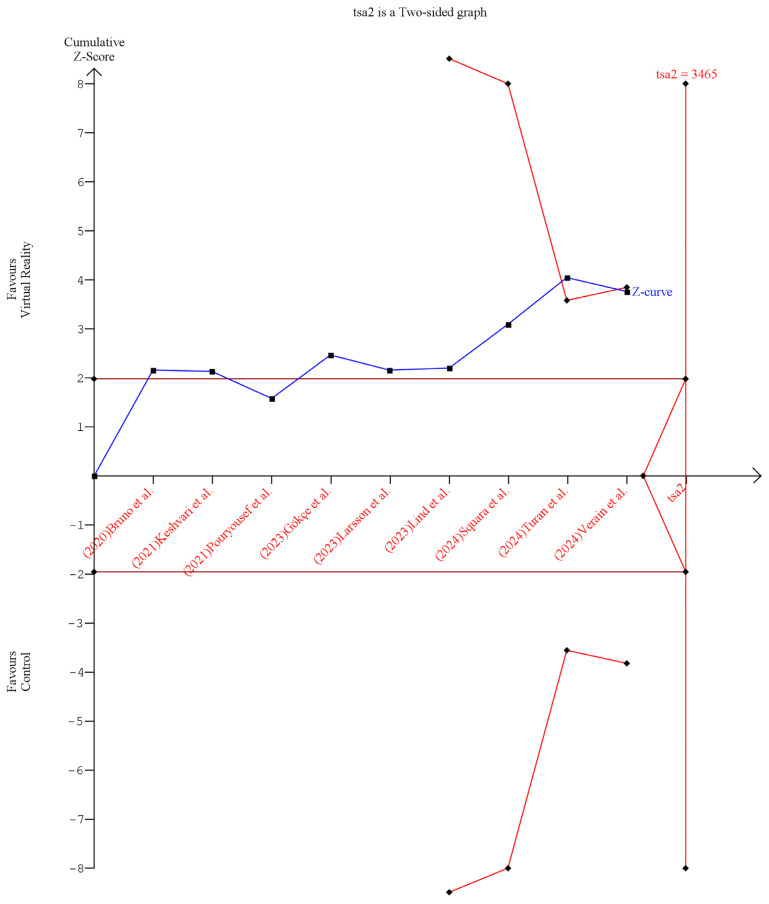
Trial sequential analysis of peri-procedure anxiety [18,20,21,22,23,25,26,27,28].

The Galbraith plot showed that four studies [20,21,22,27] are outliers and potentially responsible for the observed heterogeneity in peri-procedural anxiety, as shown in Figure S3, and three studies [21,27,28] are outliers and potentially responsible for the observed heterogeneity in peri-procedural pain, as shown in Figure S4. The test for subgroup analysis was insignificant based on distraction timing in peri-procedural anxiety (*p* = 0.97), Figure S5. However, it was significant based on procedure type in peri-procedural anxiety (*p* = 0.01), as shown in Figure S6, and peri-procedural pain (*p* = 0.001), as shown in Figure S7. Finally, the TSA results revealed that the available evidence crossed the RIS and reached the trial sequential monitoring boundary, indicating robust findings. These findings strongly suggest that VR distraction can significantly ameliorate peri-procedural anxiety, as shown in Figure 4.

### 3.5. Secondary Outcomes

#### 3.5.1. Vital Signs

**Figure 5:**
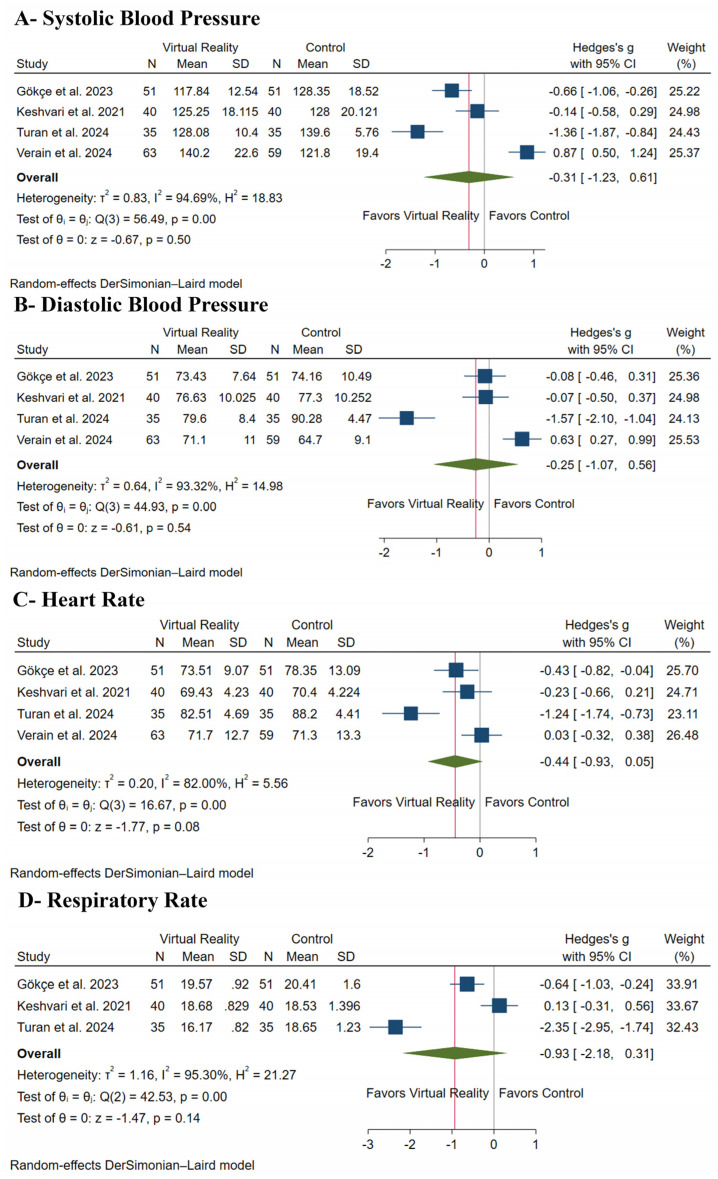
Forest plots of vital signs, CI: confidence interval [21,25,27,28].

There was no difference between both groups regarding SBP (SMD: −0.31, with 95% CI [−1.23, 0.61], *p* = 0.50) (Figure 5A), DBP (SMD: −0.25, with 95% CI [−1.07, 0.56], *p* = 0.54) (Figure 5B), HR (SMD: −0.44, with 95% CI [−0.93, 0.05], *p* = 0.08) (Figure 5C), and RR (SMD: −0.93, with 95% CI [−2.18, 0.31], *p* = 0.14) (Figure 5D). Pooled studies were heterogeneous in SBP (I^2^ = 95%, *p* < 0.001), DBP (I^2^ = 93%, *p* < 0.001), HR (I^2^ = 82%, *p* < 0.001), and RR (I^2^ = 95%, *p* < 0.001).

Leave-one-out sensitivity analysis showed consistent results in each scenario in SBP (Figure S8), DBP (Figure S10), and RR (Figure S14); however, VR distraction significantly decreased HR after excluding Verain et al. [27] (SMD: −0.61, with 95% CI [−1.17, −0.06], *p* = 0.03) (Figure S12). The Galbraith plot showed that three studies [21,27,28] are outliers and potentially responsible for the observed heterogeneity in SBP (Figure S9), two studies [21,27] in DBP and HR (Figures S11 and S13), and another two studies in RR (Figure S15).

#### 3.5.2. Procedure Duration

There was no difference between both groups regarding (SMD: 0.07, with 95% CI [−1.14, 0.28], *p* = 0.49), as shown in Figure S16. Pooled studies were homogenous (I^2^ = 0%, *p* = 0.65).

### 3.6. Safety Outcomes

VR distraction was well-tolerable; only four studies assessed the incidence of adverse events [22,24,26,27], reporting no difference between VR and control. Also, there was no difference between both groups regarding the incidence of delirium (RR: 0.98, with 95% CI [0.37, 2.63], *p* = 0.97), as shown in Figure S17. Pooled studies were homogenous (I^2^ = 0%, *p* = 0.94).

## 4. Discussion

After pooling 10 RCTs and 890 patients, VR significantly reduced peri-procedure anxiety with no effect on peri-procedure pain, vital signs, or procedure duration. VR was also well-tolerable, with no significant increase in adverse events or delirium. Experiencing high levels of peri-procedure anxiety with PCIs deteriorates patient outcomes, inducing endothelial dysfunction, increasing pain, delaying recovery, and subsequently increasing costs [37,38,39]. Patient anxiety is correlated with apprehension regarding potential complications, insufficient procedural comprehension, and inadequate patient education [4,40].

Moreover, the GRADE assessment rated the certainty of evidence as very low for certain outcomes. This was primarily due to small sample sizes, high heterogeneity, and the open-label design commonly employed across the included trials. The meta-analysis also revealed a considerable degree of heterogeneity, which may be attributed to several factors, including potential co-interventions such as variable sedation protocols, inconsistencies in procedural techniques, and differences in the type and content of virtual reality interventions. Additionally, variability in patient characteristics—such as baseline anxiety levels, comorbidities, and prior procedural experiences—may have further contributed to this heterogeneity.

Virtual reality’s substantial anxiolytic effects alleviate patient distress by altering their perception of time within negative environments and offering a more positive sensory experience [8,41,42]. VR engages users’ senses, diverting them from the central nervous system’s prosaic functions through sensory stimulation and motivational pathways [21]. Also, while using the VR glasses, a relaxing effect can be achieved by integrating calming soundscapes or music specifically selected to complement the viewed content [8]. Utilizing this method, patients redirect their attention from the external environment, consequently reducing the neural processing of pain and anxiety, alleviating pain and anxiety perceptions [43]. However, our findings were insignificant regarding the VR effect on pain.

This suggests a potential need for more extensive relaxation or distraction techniques to alleviate pain during PCI procedures, exceeding the levels typically required for anxiety management within the same context. Also, VR significantly reduced peri-procedure pain after excluding Verain et al. [27], who used conscious sedation as a control. This may indicate that conscious sedation remains more effective than VR in managing peri-procedure pain. Nevertheless, in the same study, there was no difference between both groups regarding peri-procedure anxiety [27]. Therefore, VR can be enhanced to target pain alleviation in future studies, considering the most effective content and duration.

Furthermore, the administration of analgesics or opioids during or after PCIs could successfully alleviate any pain experienced, or the PCIs may not have been painful to begin with [1], given recent advancements in interventional cardiology and most procedures are currently conducted through the trans-radial approach [15]. The incidence of serious adverse events from sedation is low [44]; however, minor side effects are observed almost daily. Instances of drowsiness, confusion, and anterograde amnesia commonly lead to the deferral of same-day discharge and can necessitate additional diagnostic procedures, including neuroimaging studies [27]. The avoidance of sedative drugs offers several notable benefits, including reduced costs, improved supply chain stability, decreased reliance on anesthesiologists, and a lower incidence of adverse effects [27]. Therefore, more research is required to determine precisely which PCI would most benefit from the integration of VR technology and to identify the optimal points within the patient’s treatment pathway for its implementation.

Moreover, the experience of pain and anxiety triggers the release of corticotropin-releasing hormone, subsequently activating the locus coeruleus, which releases noradrenaline for the rapid activation of sympathetic fibres [21]. The sympathetic nervous system subsequently triggers the release of adrenomedullary catecholamines, leading to increased heart rate, blood pressure, and respiration [45,46]. However, our results did not show that VR controlled vital signs. This can be explained by the fact that only four trials were included in the analysis of the vitals, compared to nine trials in peri-procedure anxiety. In patients undergoing PCIs, a rise in blood pressure may cause a corresponding increase in intravascular pressure, consequently increasing puncture-site complications (bleeding, hematoma, and ecchymosis), besides stroke in susceptible patients [47,48]. Therefore, more data are required before a definitive conclusion on VR’s effect on vital signs during PCIs.

Safety is another pertinent consideration before VR application. The included trials showed that VR was well-tolerable, with no significant increase in adverse events and a low incidence of cybersickness, nausea, and vomiting during VR intervention in the supine position [49]. Cybersickness is a consequence of sensory signal discrepancies; it occurs when visual motion information is perceived without congruent vestibular confirmation [50]. In this case, presenting static rather than moving images could minimize conflicting sensory information [18]. Also, the use of static imagery may prove beneficial in reducing head movement, a factor that can be problematic for individuals undergoing PCIs [18]. However, this requires further confirmation.

To the extent of our knowledge, this is the most extensive systematic review and meta-analysis investigating VR’s efficacy in mitigating pain in patients undergoing PCIs. We also conducted a thorough analysis, including leave-one-out sensitivity analysis, subgroup analysis, and TSA, along with an extensive GRADE certainty of evidence evaluation. Still, our findings are limited by the following: first, all included trials were single-centre trials with a relatively small sample size, which can affect the generalizability of our findings. Second, most outcomes showed significant heterogeneity, an inherited limitation, as the VR effect is affected by several study characteristics, including VR technical quality, patient characteristics, and procedure type. VR distraction therapy incorporates several technical tools, leading to a wide range of possible outcomes [1]; however, we investigated the sources of heterogeneity the best we could, providing a thorough heterogeneity assessment.

Third, all included trials showed at least some concerns of bias, if not a high risk of bias. This is mainly due to the open-label interventions, especially when sedation is offered, as operators may administer more sedation, ensuring enough comfort and preventing pain and anxiety regardless of the VR effect. Fourth, using multiple assessment tools, the open-label assessment of subjective outcomes, such as pain and anxiety. This can cast doubts on the reliability and generalizability of our results. Finally, after the previously mentioned limitation, the GRADE interpretation of our results was mostly very low, warranting caution.

Future large-scale trials remain warranted to confirm our findings, especially on pain, considering the following: first, future studies may consider using a more objective way of assessing pain and anxiety through a standardized measurement of vital signs to eliminate the risk of detection bias. Second, a rigorous cost–benefit analysis of VR for pain and anxiety management is necessary. This in-depth evaluation should consider the complete picture, encompassing both the direct expenses related to software and hardware procurement and the indirect costs, notably the considerable time investment required to effectively integrate VR devices into the daily routines of clinical practice [1]. Third, all future trials should report a complete description of the VR intervention, including content, duration, and timing of intervention, with a rigorous assessment of adverse events. This can be conducted following the Tidier checklist [51]. Fourth, future trials must consider the effect of VR on analgesia or sedation consumption through a clear report of the sedation used throughout and after PCIs. Finally, it would be of interest to explore the impact of patient age on the feasibility and effectiveness of VR interventions, as current studies include populations ranging widely in age—from middle-aged adults to octogenarians—who may differ in their response and adaptability to VR use during procedures.

## 5. Conclusions

VR significantly ameliorated peri-procedure anxiety in patients undergoing PCIs; however, it had no effect on peri-procedure pain or vital signs. This is based on uncertain evidence from heterogeneous studies, warranting further confirmation through large-scale RCTs.

## Figures and Tables

**Table 1:** Summary of the key characteristics of the included randomized controlled trials.

Study ID	Study Design	Country	Sample Size	Procedure	Visual Content	VR Device	VR Duration	Distraction Timing	Control	Pain Assessment Tool	Anxiety Assessment Tool	Sedation
Bruno et al. 2020 [26]	Single-centre, open-label RCT	Germany	32	TAVR	Patients could choose one of the following videos: nature scenery, an aquarium, flying over a green landscape, diving underwater, or walking through a calm forest	MEDION^®^ ERAZER^®^ X1000 MR Glasses, 7.34 cm (2.89″) LC-Display (Medion AG, Essen, Germany)	30.5 min (median)	Peri-procedure	Usual care	VAS-P (0–10)	VAS-A (0–10)	1 mg lorazepam or 3.25 mg midazolam
Gökçe et al. 2023 [28]	Single-centre, single-blinded RCT	Turkey	102	CAG	Three different image types, including a coastal forest view, an undersea view, and an open-air museum tour, with nature sound effects and relaxing background music	Oculus Virtual Reality Glasses	30 min	Peri-procedure	Usual care	VAS-P (0–10)	STAI	NA
Keshvari et al. 2021 [25]	Single-centre, open-label RCT	Iran	80	CAG	Natural scene that was filmed at various natural locations and landscapes such as the beach, mountains, waterfalls, and rivers with pleasant sounds	Remix video headset and a Huawei mobile phone	5 min	Pre-procedure	Usual care	NA	STAI	NA
Larsson et al. 2023 [23]	Single-centre, open-label RCT	France	156	CAG	Five themes were proposed to the patient (Zen Garden, forest, mountain, beach, or diving)	The Healthy Mind company (Company, Ville, Pays, Paris, France) provided the study materials (2 headsets of VR and audio headphones)	About 20 min	Pre-procedure	Usual care	NA	VAS-A (0–10)	NA
Lind et al. 2023 [22]	Single-centre, open-label RCT	Germany	117	TAVR	Different categories, e.g., nature and relaxation, travel, documentations, Hollywood movies, classical concerts	Happy Med video glasses (Happy Med GmbH, Vienna, Austria)	NA	Peri-procedure	Usual care	VAS-P (0–10)	STAI	No sedation
Pitts et al. 2024 [24]	Single-centre, open-label RCT	Germany	90	TAVR	NA	Happy Med video glasses (Happy Med GmbH, Vienna, Austria)	NA	Peri-procedure	Usual care	NRS (0–100)	STAI	10 mg of propofol 1% were given if patients could not be adequately sedated through titration of the remifentanil dose to a maximum of 0.08 mg/kg/min
Pouryousef et al. 2021 [20]	Single-centre, single-blinded RCT	Iran	60	CAG	Calming images	NA	5 min	Pre-procedure	Usual care	NA	STAI	NA
Squara et al. 2024 [18]	Single-centre, open-label RCT	France	61	ICD implantation	Static landscapes: river delta, rural India, Spitzberg, mountains in summer, or mountains in winter. Every 5 min, the video recording evolved to another static point-of-view of the chosen landscape	Deepsen (Lyon, France)	NA	Peri-procedure	Usual care	NRS (0–10)	NRS (0–10)	Intravenous paracetamol (1 g) 60 min before the procedure
Turan et al. 2024 [21]	Single-centre, open-label RCT	Turkey	70	CAG	The licenced product “Secret Garden”	An android mobile phone placed in the Cardboard Super Flex Binoculars Glasses	30–45 min	Peri-procedure	Usual care	VAS-P (0–10)	Anxiety Assessment Scale (AAS)	NA
Verain et al. 2024 [27]	Single-centre, open-label RCT	France	122	CAG, coronary angioplasty, or peripheral angioplasty	NA	Deepsen (Lyon, France)	NA	Peri-procedure	Sedation	VAS-P (0–10)	STAI	Midazolam and fentanyl

CAG, coronary angiography; ICD, implantable cardiac defibrillator; STAI, state-trait anxiety inventory; TAVR, transcatheter aortic valve replacement; NA, not available; VR, virtual reality; VAS-P, visual analogue scale—pain; VAS-A, visual analogue scale—anxiety.

**Table 2:** Baseline characteristics of participants in the included randomized controlled trials.

Study ID	Number of Patients in Each Group		Age (Years)		Gender (Male)		BMI		Comorbidities											
	VR	Control	VR	Control	VR	Control	VR	Control	Heart Failure		COPD		CAD		DM		HTN		Smoking	
									VR	Control	VR	Control	VR	Control	VR	Control	VR	Control	VR	Control
Bruno et al. 2020 [26]	16	16	82 (78.3–87)	83 (78.3–86.8)	11 (68.8)	9 (56.3)	NA	NA	9 (56.3)	9 (56.3)	6 (37.5)	4 (25)	11 (68.8)	14 (87.5)	6 (37.5)	4 (25)	NA	NA	NA	NA
Gökçe et al. 2023 [28]	51	51	59.4 ± 12.1	58.2 ± 12.0	25 (49)	31 (60.8)	28.5 ± 5.9	27.3 ± 4.5	NA	NA	NA	NA	NA	NA	NA	NA	NA	NA	14 (27.5)	13 (25.5)
Keshvari et al. 2021 [25]	40	40	4.002 ± 52.08	4.120 ± 50.95	32 (80)	25 (62.5)	NA	NA	NA	NA	NA	NA	NA	NA	NA	NA	NA	NA	NA	NA
Larsson et al. 2023 [23]	76	80	62.5 ± 10.9	62.6 ± 9.5	56(73.7)	55(68.7)	27.3 ± 5.3	27.8 ± 5.2	NA	NA	NA	NA	NA	NA	16 (21)	22 (27.5)	37 (48.7)	34 (42.5)	14(18.4)	15(18.7)
Lind et al. 2023 [22]	59	58	81.1 ± 5.7	81.2 ± 5.5	30 (50.8)	29 (50.0)	27.3 ± 4.3	26.3 ± 4.3	48 (81.4)	42 (72.4)	NA	NA	41 (69.5)	34 (58.6)	16 (29.1)	17 (32.1)	NA	NA	NA	NA
Pitts et al. 2024 [24]	45	45	79 (76–83)	81 (76–84)	32 (77)	19 (42)	28.3 (24.9–30.6)	28.1 (23.8–30.1)	NA	NA	7 (16)	5 (11)	34 (76)	31(69)	12 (27)	15 (33)	39 (87)	44 (98)	NA	NA
Pouryousef et al. 2021 [20]	30	30	49.96 ± 8.10	51.36 ± 8.11	NA	NA	NA	NA	NA	NA	NA	NA	NA	NA	12 (40)	13 (43.3)	NA	NA	NA	NA
Squara et al. 2024 [18]	30	31	78.3 ± 8.4	77.5 ± 8.0	21 (70)	17 (54.5)	NA	NA	NA	NA	NA	NA	NA	NA	13 (43.3)	11 (35.5)	23 (76.7)	21 (67.8)	NA	NA
Turan et al. 2024 [21]	35	35	70.00 ± 9.82	65.20 ± 13.57	16 (45.7)	18 (51.4)	NA	NA	NA	NA	NA	NA	NA	NA	NA	NA	NA	NA	NA	NA
Verain et al. 2024 [27]	63	59	68.5 ± 10.0	68.8 ± 9.5	50 (79.4)	42 (71.2)	28.2 ± 5.4	29.0 ± 5.0	NA	NA	NA	NA	NA	NA	22 (34.9)	20 (33.9)	38 (60.3)	43 (72.9)	NA	NA

Continuous data are presented in mean ± standard deviation or median (interquartile range); dichotomous data are presented in number of patients (%); NA, not available; BMI, body mass index; CAD, coronary artery disease; COPD, chronic obstructive pulmonary disease; DM, diabetes mellitus; HTN, hypertension; VR, virtual reality.

**Table 3:** Evidence profile summarizing the certainty of evidence for each outcome based on the Grading of Recommendations Assessment, Development, and Evaluation (GRADE) approach.

Certainty Assessment							Summary of Findings				
Participants (Studies) Follow-Up	Risk of Bias	Inconsistency	Indirectness	Imprecision	Publication Bias	Overall Certainty of Evidence	Study Event Rates (%)		Relative Effect (95% CI)	Anticipated Absolute Effects	
							With [Control]	With [VR]		Risk with [Control]	Risk Difference with [VR]
Peri-procedural Anxiety											
783 (9 RCTs)	serious ^a^	very serious ^b^	not serious	serious ^c^	none	⨁◯◯◯ Very low ^a,b,c^	397	386	-	-	SMD 0.7 SD lower (1.15 lower to 0.26 lower)
Peri-procedural Pain											
504 (6 RCTs)	serious ^a^	very serious ^b^	not serious	serious ^c^	none	⨁◯◯◯ Very low ^a,b,c^	250	254	-	-	SMD 0.64 SD lower (1.45 lower to 0.16 higher)
Systolic Blood Pressure											
374 (4 RCTs)	serious ^a^	very serious ^b^	not serious	serious ^c^	none	⨁◯◯◯ Very low ^a,b,c^	185	189	-	-	SMD 0.31 SD lower (1.23 lower to 0.61 higher)
Diastolic Blood Pressure											
374 (4 RCTs)	serious ^a^	very serious ^c^	not serious	serious ^c^	none	⨁◯◯◯ Very low ^a,c^	185	189	-	-	SMD 0.25 SD lower (1.07 lower to 0.56 higher)
Heart Rate											
374 (4 RCTs)	serious ^a^	very serious ^c^	not serious	serious ^c^	none	⨁◯◯◯ Very low ^a,c^	185	189	-	-	SMD 0.44 SD lower (0.93 lower to 0.05 higher)
Respiratory Rate											
252 (3 RCTs)	serious ^a^	very serious ^b^	not serious	serious ^c^	none	⨁◯◯◯ Very low ^a,b,c^	126	126	-	-	SMD 0.93 SD lower (2.18 lower to 0.31 higher)
Procedure Duration											
346 (3 RCTs)	serious ^a^	not serious	not serious	not serious	none	⨁⨁⨁◯ Moderate ^a^	180	166	-	-	SMD 0.07 SD higher (0.14 lower to 0.28 higher)
Delirium											
361 (4 RCTs)	serious ^a^	not serious	not serious	serious ^c^	none	⨁⨁◯◯ Low ^a,c^	7/178 (3.9%)	7/183 (3.8%)	RR 0.98 (0.37 to 2.63)	7/178 (3.9%)	1 fewer per 1000 (from 25 fewer to 64 more)

CI: confidence interval; RR: risk ratio; SMD: standardized mean difference; Explanations: ^a^ All trials had at least some concerns of overall bias, ^b^ I^2^ > 75%, ^c^ A wide confidence interval that does not exclude the appreciable risk of harm/benefit.

## Data Availability

All data are available within the manuscript and can be obtained from the corresponding author upon reasonable request.

## Supplementary Materials

The following supporting information can be downloaded at: https://www.mdpi.com/article/10.3390/medicina61060957/s1, Figure S1: Leave-one-out sensitivity analysis of peri-procedural anxiety; Figure S2: Leave-one-out sensitivity analysis of peri-procedural pain; Figure S3: Galbraith plot of peri-procedural anxiety; Figure S4: Galbraith plot of peri-procedural pain; Figure S5: Subgroup analysis based on distraction timing in peri-procedural anxiety; Figure S6: Subgroup analysis based on procedure type in peri-procedural anxiety; Figure S7: Subgroup analysis based on procedure type in peri-procedural pain; Figure S8: Leave-one-out sensitivity analysis of systolic blood pressure; Figure S9: Galbraith plot of systolic blood pressure; Figure S10: Leave-one-out sensitivity analysis of diastolic blood pressure; Figure S11: Galbraith plot of diastolic blood pressure; Figure S12: Leave-one-out sensitivity analysis of heart rate; Figure S13: Galbraith plot of heart rate; Figure S14: Leave-one-out sensitivity analysis of respiratory rate; Figure S15: Galbraith plot of respiratory rate; Figure S16: Forest plot of procedure duration; Figure S17: Forest plot of delirium; Table S1: Search strategy; Table S2: Excluded records during full-text screening.

## References

[1] El Mathari S., Hoekman A., Kharbanda R.K., Sadeghi A.H., de Lind van Wijngaarden R., Götte M., Klautz R.J., Kluin J. (2024). Virtual Reality for Pain and Anxiety Management in Cardiac Surgery and Interventional Cardiology. JACC Adv..

[2] Younes O., Amer R., Fawzy H., Shama G. (2019). Psychiatric disturbances in patients undergoing open-heart surgery. Middle East Curr. Psychiatry.

[3] Ayasrah S.M., Ahmad M.M. (2016). Educational video intervention effects on periprocedural anxiety levels among cardiac catheterization patients: A randomized clinical trial. Res. Theory Nurs. Pract..

[4] Delewi R., Vlastra W., Rohling W.J., Wagenaar T.C., Zwemstra M., Meesterman M.G., Vis M.M., Wykrzykowska J.J., Koch K.T., de Winter R.J. (2017). Anxiety levels of patients undergoing coronary procedures in the catheterization laboratory. Int. J. Cardiol..

[5] Choinière M., Watt-Watson J., Victor J.C., Baskett R.J., Bussières J.S., Carrier M., Cogan J., Costello J., Feindel C., Guertin M.-C. (2014). Prevalence of and risk factors for persistent postoperative nonanginal pain after cardiac surgery: A 2-year prospective multicentre study. CMAJ Can. Med. Assoc. J..

[6] Guimarães-Pereira L., Reis P., Abelha F., Azevedo L.F., Castro-Lopes J.M. (2017). Persistent postoperative pain after cardiac surgery: A systematic review with meta-analysis regarding incidence and pain intensity. Pain.

[7] Miozzo A.P., Stein C., Bozzetto C.B., Plentz R.D.M. (2016). Massage therapy reduces pain and anxiety after cardiac surgery: A systematic review and meta-analysis of randomized clinical trials. Clin. Trials Regul. Sci. Cardiol..

[8] Saab O., Al-Obaidi H., Merza N., Bhagat U., Al-Sagban A., Algodi M., Abuelazm M., El-Serag H. (2024). The Impact of Visual Distraction Interventions on Patients’ Pain and Anxiety During Colonoscopy: A Systematic Review and Meta-Analysis of Randomized Controlled Trials. J. Clin. Gastroenterol..

[9] Varon J., Marik P.E. (2008). Perioperative hypertension management. Vasc. Health Risk Manag..

[10] Goldberg M.E., Weaver F.A. (2007). Strategies for managing perioperative hypertension. Crit. Care Clin..

[11] Baytar A.A., Bollucuo K. (2023). Effect of virtual reality on preoperative anxiety in patients undergoing septorhinoplasty. Braz. J. Anesthesiol. (Engl. Ed.).

[12] Hughes O., MacQuhae F., Rakosi A., Herskovitz I., Kirsner R.S. (2016). Stress and wound healing. Stress and Skin Disorders.

[13] Munafò M.R., Stevenson J. (2001). Anxiety and surgical recovery. J. Psychosom. Res..

[14] Sloot S., Boland J., Snowden J.A., Ezaydi Y., Foster A., Gethin A., Green T., Chopra L., Verhagen S., Vissers K. (2015). Side effects of analgesia may significantly reduce quality of life in symptomatic multiple myeloma: A cross-sectional prevalence study. Support. Care Cancer.

[15] Abdelazeem B., Abuelazm M.T., Swed S., Gamal M., Atef M., Al-Zeftawy M.A., Noori M.A., Lutz A., Volgman A.S. (2022). The efficacy of nitroglycerin to prevent radial artery spasm and occlusion during and after transradial catheterization: A systematic review and meta-analysis of randomized controlled trials. Clin. Cardiol..

[16] Deftereos S., Giannopoulos G., Raisakis K., Hahalis G., Kaoukis A., Kossyvakis C., Avramides D., Pappas L., Panagopoulou V., Pyrgakis V. (2013). Moderate procedural sedation and opioid analgesia during transradial coronary interventions to prevent spasm: A prospective randomized study. JACC Cardiovasc. Interv..

[17] Apfelbaum J., Gross J.B., Connis R.T., Agarkar M., Arnold D.E., Coté C.J., Tung A. (2018). Practice Guidelines for Moderate Procedural Sedation and Analgesia 2018: A Report by the American Society of Anesthesiologists Task Force on Moderate Procedural Sedation and Analgesia, the American Association of Oral and Maxillofacial Surgeons, American College of Radiology, American Dental Association, American Society of Dentist Anesthesiologists, and Society of Interventional Radiology. Anesthesiology.

[18] Squara F., Bateau J., Scarlatti D., Bun S.-S., Moceri P., Ferrari E. (2024). Virtual Reality for the Management of Pain and Anxiety in Patients Undergoing Implantation of Pacemaker or Implantable Cardioverter Defibrillator: A Randomized Study. J. Med. Syst..

[19] Indovina P., Barone D., Gallo L., Chirico A., De Pietro G., Giordano A. (2018). Virtual Reality as a Distraction Intervention to Relieve Pain and Distress during Medical Procedures. Clin. J. Pain.

[20] Pouryousef F., Navidian A., Ghahdarijani O.R., Yaghoubinia F. (2021). Comparing the Effect of Virtual Reality and Rhythmic Breathing on the Anxiety of the Patients Undergoing Coronary Angiography. Q. Horiz. Med. Sci..

[21] Turan G.B., Gür F., Özer Z., Tarkan Ç. (2024). Effects of Virtual Reality on Pain, Anxiety, Patient Satisfaction in Coronary Angiography: A Randomized Trial. Pain Manag. Nurs..

[22] Lind A., Ahsan M., Totzeck M., Al-Rashid F., Haddad A., Dubler S., Brenner T., Skarabis A., El Gabry M., Rassaf T. (2023). Virtual reality-assisted distraction during transcatheter aortic valve implantation under local anaesthesia: A randomised study. Int. J. Cardiol..

[23] Larsson C.E., Cabassut V., Peretout P., Marliere S., Vautrin E., Piliero N., Salvat M., Riou L., Vanzetto G., Vilotitch A. (2023). Assessment of the Objective Effect of Virtual Reality for Preoperative Anxiety in Interventional Cardiology. Am. J. Cardiol..

[24] Pitts L., Hellner N., Kofler M., Ryschka M., Unbehaun A., O’Brien B., Kempfert J., Hommel M. (2024). The Influence of Audiovisual Distraction on Pain Reduction During Transcatheter Aortic Valve Implantation Under Monitored Anesthesia Care: A Prospective Randomized Trial. J. Cardiothorac. Vasc. Anesth..

[25] Keshvari M., Yeganeh M.R., Paryad E., Roushan Z.A., Pouralizadeh M. (2021). The effect of virtual reality distraction on reducing patients’ anxiety before coronary angiography: A randomized clinical trial study. Egypt. Heart J..

[26] Bruno R.R., Lin Y., Wolff G., Polzin A., Veulemans V., Klein K., Westenfeld R., Zeus T., Kelm M., Jung C. (2020). Virtual reality-assisted conscious sedation during transcatheter aortic valve implantation: A randomised pilot study. EuroIntervention.

[27] Verain J., Trouillet C., Moulin F., Christophe C. (2024). Efficacy of virtual reality therapy versus pharmacological sedation for reducing pain and anxiety during coronary catheterisation procedures: A prospective randomised controlled trial. Health Sci. Rep..

[28] Gökçe E., Arslan S. (2023). Effects of virtual reality and acupressure interventions on pain, anxiety, vital signs and comfort in catheter extraction processes for patients undergoing coronary angiography: A randomized controlled trial. Int. J. Nurs. Pract..

[29] Page M.J., McKenzie J.E., Bossuyt P.M., Boutron I., Hoffmann T.C., Mulrow C.D., Shamseer L., Tetzlaff J.M., Akl E.A., Brennan S.E. (2021). The The PRISMA 2020 statement: An updated guideline for reporting systematic reviews. Syst. Rev..

[30] Higgins J.P.T., Thomas J., Chandler J., Cumpston M., Li T., Page M.J., Welch V. (2023). Cochrane Handbook for Systematic Reviews of Interventions.

[31] Wan X., Wang W., Liu J., Tong T. (2014). Estimating the sample mean and standard deviation from the sample size, median, range and/or interquartile range. BMC Med. Res. Methodol..

[32] Sterne J.A.C., Savović J., Page M.J., Elbers R.G., Blencowe N.S., Boutron I., Cates C.J., Cheng H.Y., Corbett M.S., Eldridge S.M. (2019). RoB: A revised tool for assessing risk of bias in randomised trials. BMJ.

[33] Guyatt G.H., Oxman A.D., Kunz R., Vist G.E., Falck-Ytter Y., Schünemann H.J. (2008). Rating Quality of Evidence and Strength of Recommendations: What is “quality of evidence” and why is it important to clinicians?. BMJ Br. Med. J..

[34] Guyatt G.H., Oxman A.D., Vist G.E., Kunz R., Falck-Ytter Y., Alonso-Coello P., Schünemann H.J. (2008). Rating Quality of Evidence and Strength of Recommendations: GRADE: An emerging consensus on rating quality of evidence and strength of recommendations. BMJ Br. Med. J..

[35] Lin L., Chu H. (2018). Quantifying publication bias in meta-analysis. Biometrics.

[36] (2021). TSA–ctu.dk. http://ctu.dk/tsa/.

[37] Trotter R., Gallagher R., Donoghue J. (2011). Anxiety in patients undergoing percutaneous coronary interventions. Heart Lung J. Acute Crit. Care.

[38] Tadesse M., Ahmed S., Regassa T., Girma T., Hailu S., Mohammed A., Mohammed S. (2022). Effect of preoperative anxiety on postoperative pain on patients undergoing elective surgery: Prospective cohort study. Ann. Med. Surg..

[39] Liblik K., Théberge E., Gomes Z., Burbidge E., Menon N., Gobran J., Johri A.M. (2023). Improving Wellbeing After Acute Coronary Syndrome. Curr. Probl. Cardiol..

[40] Caldwell P.H., Arthur H.M., Natarajan M., Anand S.S. (2007). Fears and beliefs of patients regarding cardiac catheterization. Soc. Sci. Med..

[41] Smith V., Warty R.R., Sursas J.A., Payne O., Nair A., Krishnan S., da Silva Costa F., Wallace E.M., Vollenhoven B. (2020). The Effectiveness of Virtual Reality in Managing Acute Pain and Anxiety for Medical Inpatients: Systematic Review. J. Med. Internet Res..

[42] Seabrook E., Kelly R., Foley F., Theiler S., Thomas N., Wadley G., Nedeljkovic M. (2020). Understanding how virtual reality can support mindfulness practice: Mixed methods study. J. Med. Internet Res..

[43] Hoffman H.G., Chambers G.T., Meyer W.J., Arceneaux L.L., Russell W.J., Seibel E.J., Richards T.L., Sharar S.R., Patterson D.R. (2011). Virtual reality as an adjunctive non-pharmacologic analgesic for acute burn pain during medical procedures. Ann. Behav. Med..

[44] Doyle C., Lennox L., Bell D. (2013). A systematic review of evidence on the links between patient experience and clinical safety and effectiveness. BMJ Open.

[45] Caes L., Orchard A., Christie D. (2017). Connecting the mind–body split: Understanding the relationship between symptoms and emotional well-being in chronic pain and functional gastrointestinal disorders. Healthcare.

[46] Bushnell M.C., Ceko M., Low L.A. (2013). Cognitive and emotional control of pain and its disruption in chronic pain. Nat. Rev. Neurosci..

[47] Shoulders-Odom B. (2008). Management of patients after percutaneous coronary interventions. Crit. Care Nurse.

[48] Menekli T., Yaprak B., Doğan R. (2022). The Effect of Virtual Reality Distraction Intervention on Pain, Anxiety, and Vital Signs of Oncology Patients Undergoing Port Catheter Implantation: A Randomized Controlled Study. Pain Manag. Nurs..

[49] Gallagher M., Dowsett R., Ferrè E.R. (2019). Vection in virtual reality modulates vestibular-evoked myogenic potentials. Eur. J. Neurosci..

[50] Weech S., Kenny S., Barnett-Cowan M. (2019). Presence and cybersickness in virtual reality are negatively related: A review. Front. Psychol..

[51] Juexuan C., Yuting D., Zhaoxiang B., Chi Z., Yaolong C. (2020). Better reporting of interventions: Template for intervention description and replication (TIDieR) checklist and guide. Chin. J. Evid. Based Med..

