# Supplementary material for: Virtual Reality Distraction for Reducing Pain and Anxiety During Percutaneous Cardiovascular Interventions: A Systematic Review and Meta-Analysis with Trial Sequential Analysis
Source: Medicina (Kaunas). 2025 May 22;61(6):957. doi: 10.3390/medicina61060957 (PMC12195424; doi:10.3390/medicina61060957)
Supplement: Supplementary file 1 [file medicina-61-00957-s001.zip › medicina-3627510-supplementary.pdf]

Supplementary Materials:

Contents:

Tables.

Table S1: Search strategy.

Table S2: Excluded records during full-text screening.

Figures.

Figure S1: Leave-one-out sensitivity analysis of peri-procedural anxiety.

Figure S2: Leave-one-out sensitivity analysis of peri-procedural pain.

Figure S3: Galbraith plot of peri-procedural anxiety.

Figure S4: Galbraith plot of peri-procedural pain.

Figure S5: Subgroup analysis based on distraction timing in peri-procedural anxiety.

Figure S6: Subgroup analysis based on procedure type in peri-procedural anxiety.

Figure S7: Subgroup analysis based on procedure type in peri-procedural pain.

Figure S8: Leave-one-out sensitivity analysis of systolic blood pressure.

Figure S9: Galbraith plot of systolic blood pressure.

Figure S10: Leave-one-out sensitivity analysis of diastolic blood pressure.

Figure S11: Galbraith plot of diastolic blood pressure.

Figure S12: Leave-one-out sensitivity analysis of heart rate.

Figure S13: Galbraith plot of heart rate.

Figure S14: Leave-one-out sensitivity analysis of respiratory rate.

Figure S15: Galbraith plot of respiratory rate.

Figure S16: Forest plot of procedure duration.

Figure S17: Forest plot of delirium.

Database	Search Terms	Search Field	Search Results
PubMed	(“virtual reality” OR “smart glass*” OR “immersive” OR “non-immersive” OR “head-mounted display” OR “augmented reality” OR “mixed reality” OR “virtual therapy” OR “virtual environment” OR “virtual treatment” OR “visual distract*” OR “audiovisual distract*” OR “photic stimulation” OR “motion picture*” OR “watch* video*”) AND ("interventional cardiology" OR "cardiac intervention" OR "percutaneous cardiac procedure*" OR "percutaneous coronary intervention" OR "PCI" OR "cardiac catheter*" OR "coronary angiograph*" OR "cardiac device implant*" OR "pacemaker implant*" OR "cardiac ablation" OR "TAVR" OR "TAVI" OR "transcatheter aortic valve replacement" OR "transcatheter aortic valve implantation" OR "endovascular procedure*")	All Fields	305
Cochrane	(“virtual reality” OR “smart glass*” OR “immersive” OR “non-immersive” OR “head-mounted display” OR “augmented reality” OR “mixed reality” OR “virtual therapy” OR “virtual environment” OR “virtual treatment” OR “visual distract*” OR “audiovisual distract*” OR “photic stimulation” OR “motion picture*” OR “watch* video*”) AND ("interventional cardiology" OR "cardiac intervention" OR "percutaneous cardiac procedure*" OR "percutaneous coronary intervention" OR "PCI" OR "cardiac catheter*" OR "coronary angiograph*" OR "cardiac device implant*" OR "pacemaker implant*" OR "cardiac ablation" OR "TAVR" OR "TAVI" OR "transcatheter aortic valve replacement" OR "transcatheter aortic valve implantation" OR "endovascular procedure*")	All Text	49
WOS	(“virtual reality” OR “smart glass*” OR “immersive” OR “non-immersive” OR “head-mounted display” OR “augmented reality” OR “mixed reality” OR “virtual therapy” OR “virtual environment” OR “virtual treatment” OR “visual distract*” OR “audiovisual distract*” OR “photic stimulation” OR “motion picture*” OR “watch* video*”) AND ("interventional cardiology" OR "cardiac intervention" OR "percutaneous cardiac procedure*" OR "percutaneous coronary intervention" OR "PCI" OR "cardiac catheter*" OR "coronary angiograph*" OR "cardiac device implant*" OR "pacemaker implant*" OR "cardiac ablation" OR "TAVR" OR "TAVI" OR "transcatheter aortic valve replacement" OR "transcatheter aortic valve implantation" OR "endovascular procedure*")	All Fields	285
SCOPUS	TITLE-ABS (("virtual reality" OR "smart glass*" OR "immersive" OR "non-immersive" OR "head-mounted display" OR "augmented reality" OR "mixed reality" OR "virtual therapy" OR "virtual environment" OR "virtual treatment" OR "visual distract*" OR "audiovisual distract*" OR "photic stimulation" OR "motion picture*" OR "watch* video*") AND ("interventional cardiology" OR "cardiac intervention" OR "percutaneous cardiac procedure*" OR "percutaneous coronary intervention" OR "PCI" OR "cardiac catheter*" OR "coronary angiograph*" OR "cardiac device implant*" OR "pacemaker implant*" OR "cardiac ablation" OR "TAVR" OR "TAVI" OR "transcatheter aortic valve replacement" OR "transcatheter aortic valve implantation" OR "endovascular procedure*"))	Title, Abstract	220
Google Scholar	(“virtual reality” OR “smart glass*” OR “immersive” OR “non-immersive” OR “head-mounted display” OR “augmented reality” OR “mixed reality” OR “virtual therapy” OR “virtual environment” OR “virtual treatment” OR “visual distract*” OR “audiovisual distract*” OR “photic stimulation” OR “motion picture*” OR “watch* video*”) AND ("interventional cardiology" OR "cardiac intervention" OR "percutaneous cardiac procedure*" OR "percutaneous coronary intervention" OR "PCI" OR "cardiac catheter*" OR "coronary angiograph*" OR "cardiac device implant*" OR "pacemaker implant*" OR "cardiac ablation" OR "TAVR" OR "TAVI" OR "transcatheter aortic valve replacement" OR "transcatheter aortic valve implantation" OR "endovascular procedure*")	All Fields	97

Table S1: Search Strategy.

Title	Published Year	DOI	Study ID	Exclusion reason
Comparing Conventional Physician-Led Education with VR Education for Pacemaker Implantation: A Randomized Study.	2024	10.3390/healthcare12100976	Drozdova 2024	Wrong intervention ;
Virtual reality to reduce periprocedural anxiety during invasive coronary angiography: rationale and design of the VR InCard trial.	2024	10.1136/openhrt-2024-002628	BreunissenEHW 2024	Study protocol
The Effect of a VIrtual ReaIiTy Immersive Experience Upon Anxiety Levels, Procedural Understanding, and Satisfaction in Patients Undergoing CArdiac CaTHeterization: The VIRTUAL CATH Trial.	2021	10.25270/jic/20.00664	Morgan 2021	Wrong intervention ;
Implementation of virtual reality for patient distraction during diagnostic cardiac catheterisation.	2022	10.1017/S1047951121002845	Zablah 2022	Wrong study design;
Virtual Reality for Sedation During Atrial Fibrillation Ablation in Clinical Practice: Observational Study.	2021	10.2196/26349	Roxburgh 2021	Wrong study design;
Anxiety Reduction in TAVI using Virtual Reality Trial	2021	-	NL-OMON50951 2021	Study protocol
Efficacy of virtual reality reducing anxiety during CTO revascularization: the ReViCTO trial design	2023	10.24875/RECIC.M23000370	Fernandez-Cisnal 2023	Study protocol
Decreasing Patient Anxiety During Revascularization of Chronic Total Coronary Occlusions Using Virtual Reality Glasses	2022	-	NCT05458999 2022	Study protocol ;
the Effectiveness of Therapeutic Virtual Reality Versus Pharmacological Sedation on Pain and Anxiety During Interventional Cardiology Procedures	2022	-	NCT05588232 2022	Study protocol

Table S2: Excluded records during full-text screening.

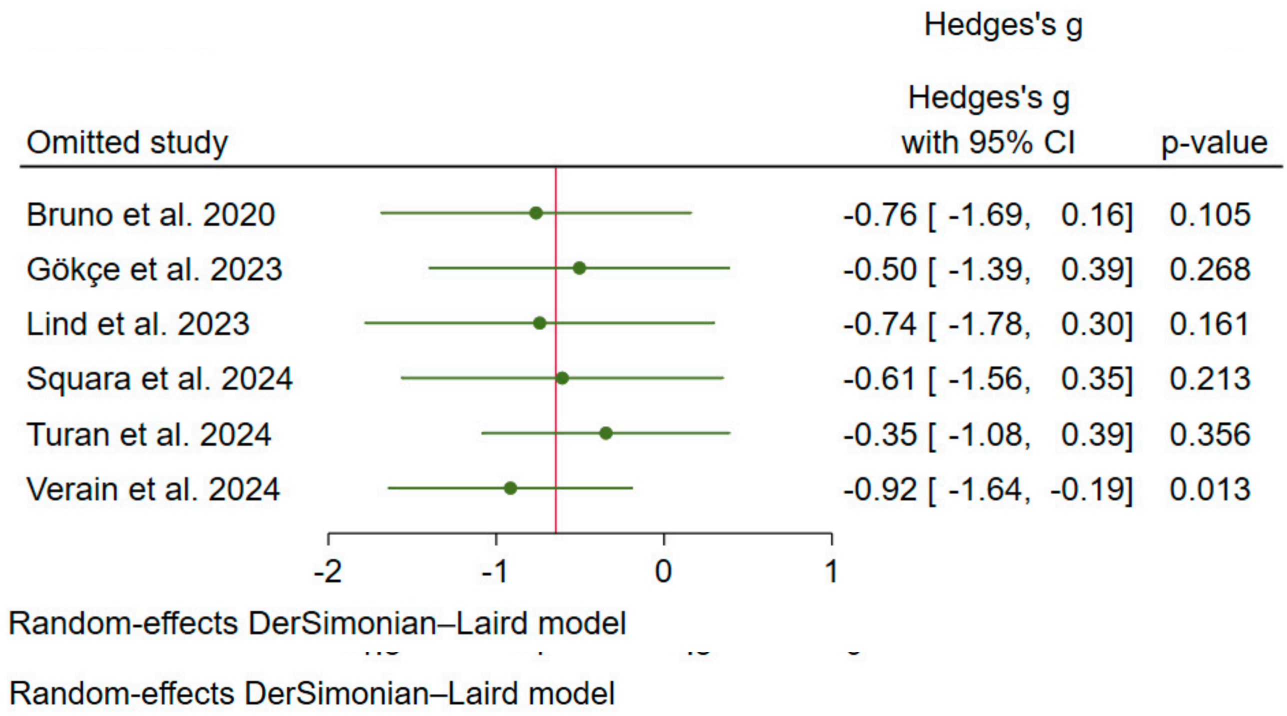

Figure S2: Leave-one-out sensitivity analysis of peri-procedural pain.

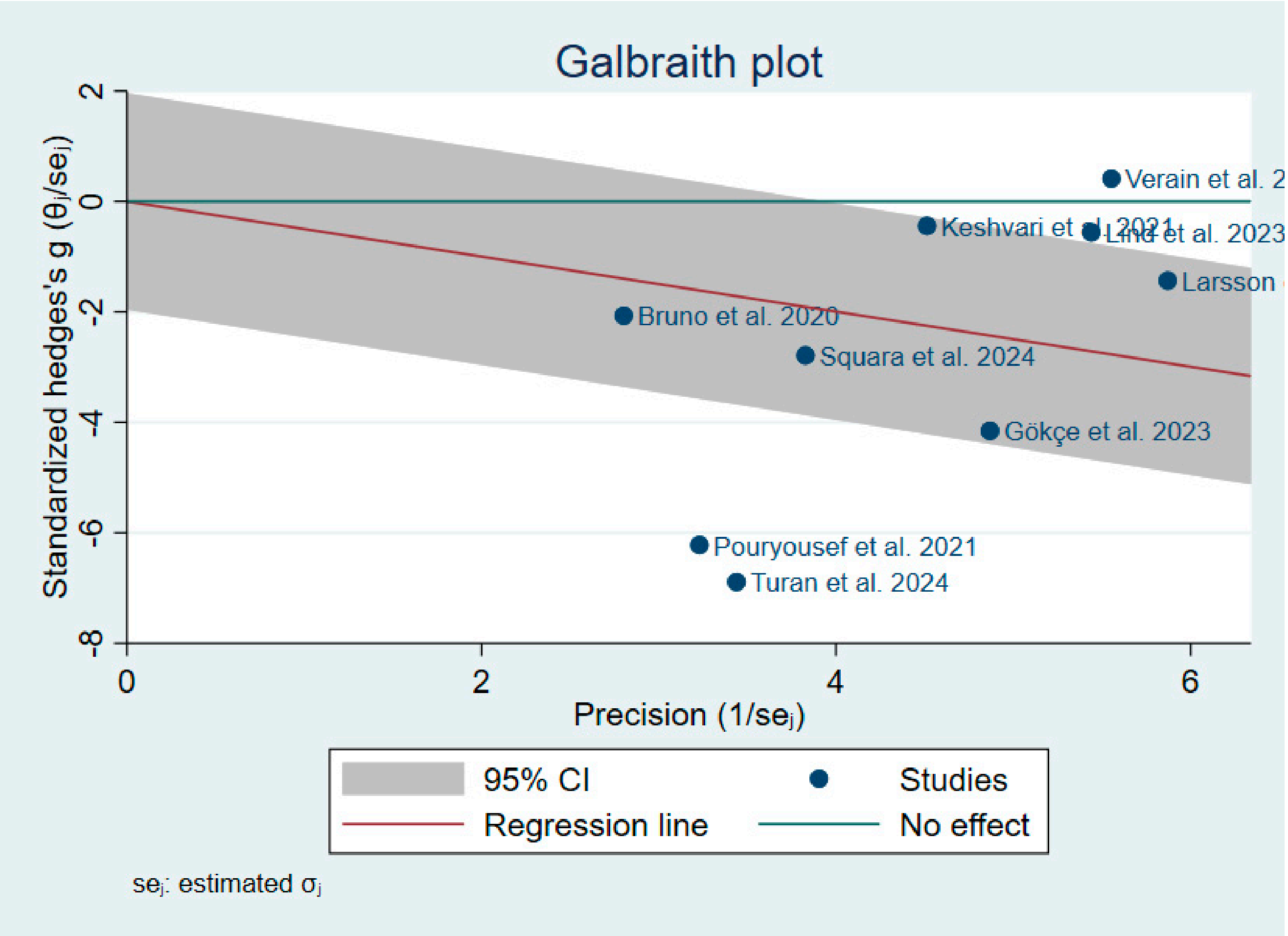

Figure S3: Galbraith plot of peri-procedural anxiety.

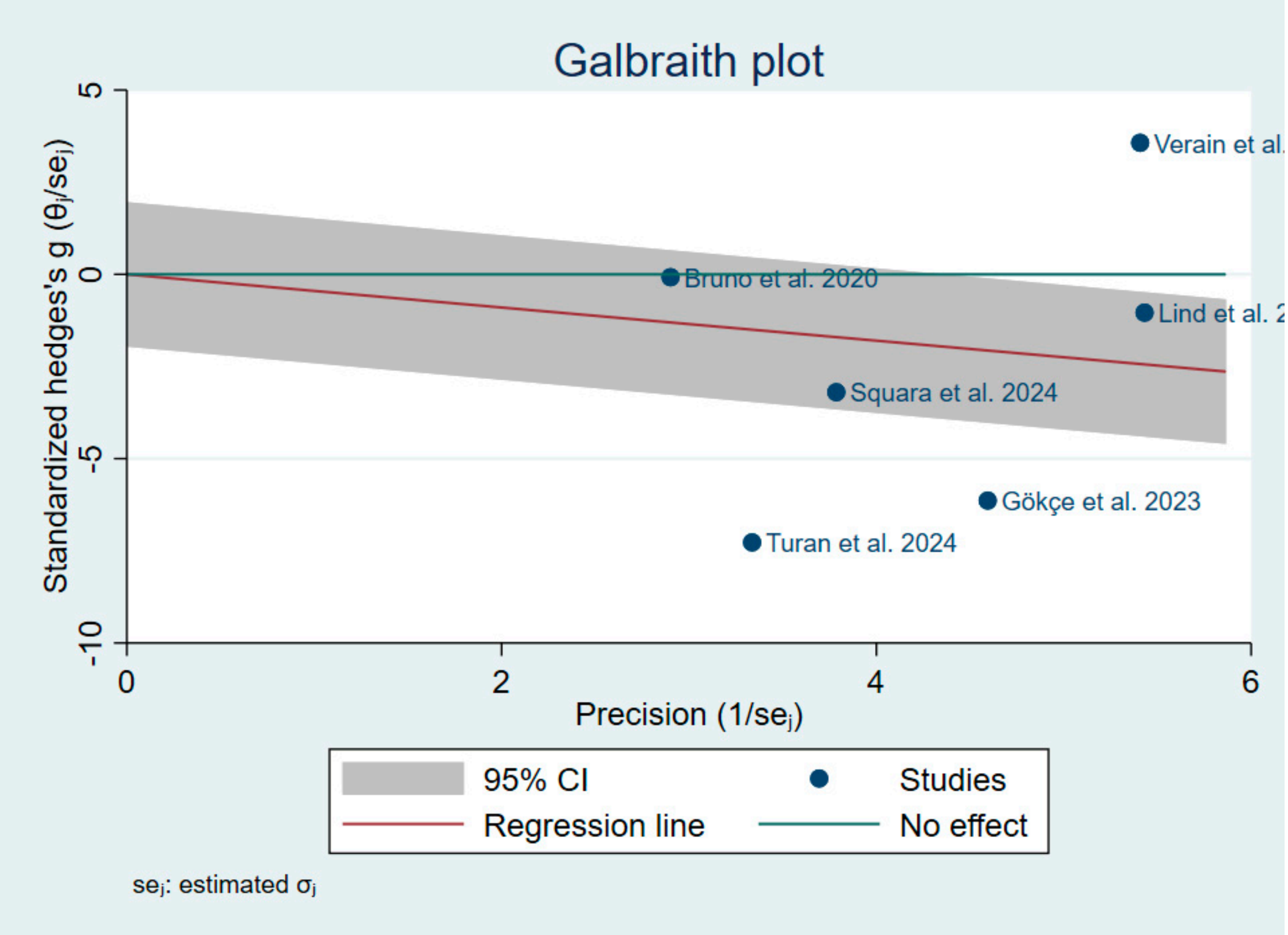

Figure S4: Galbraith plot of peri-procedural pain.

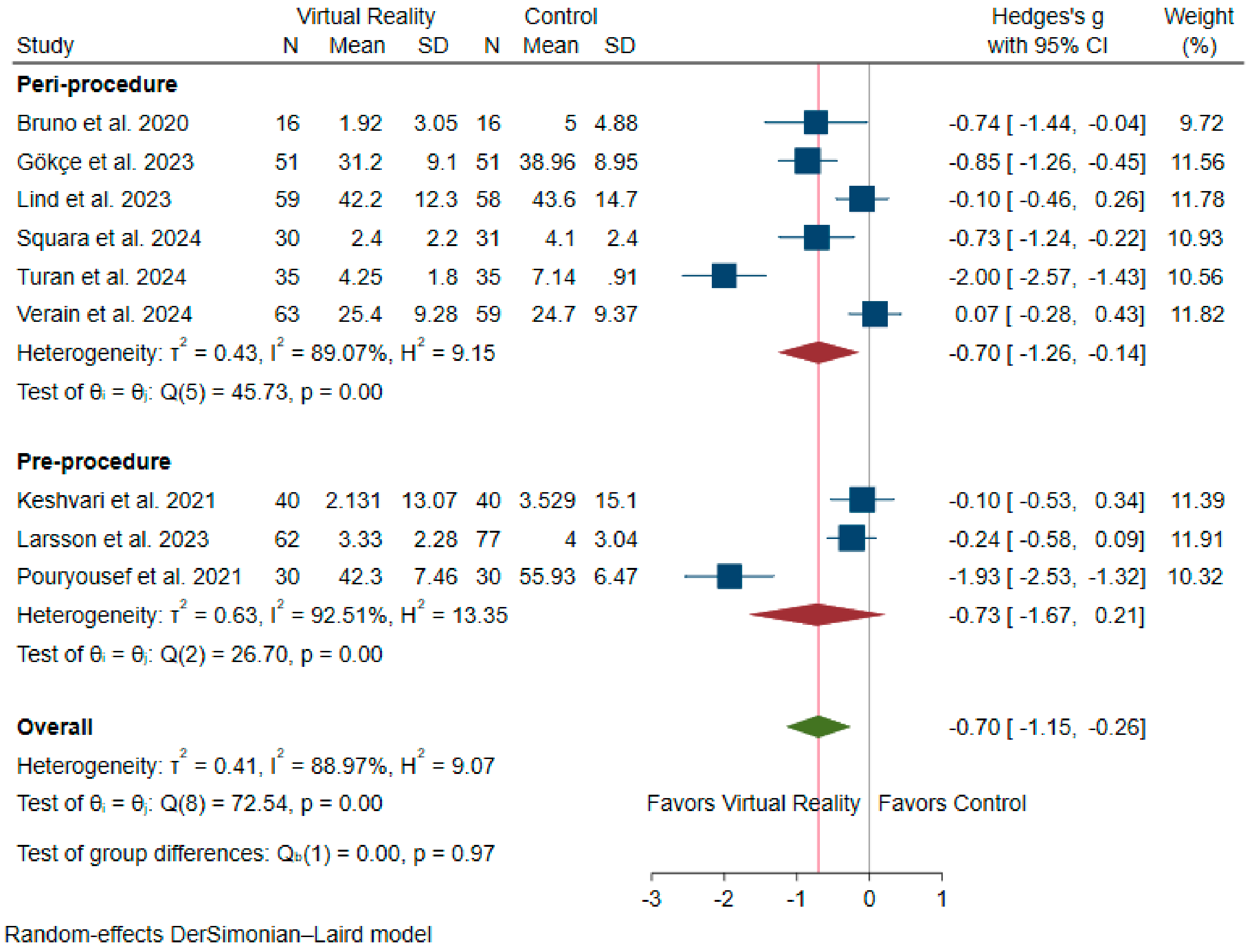

Figure S5: Subgroup analysis based on distraction timing in peri-procedural anxiety.

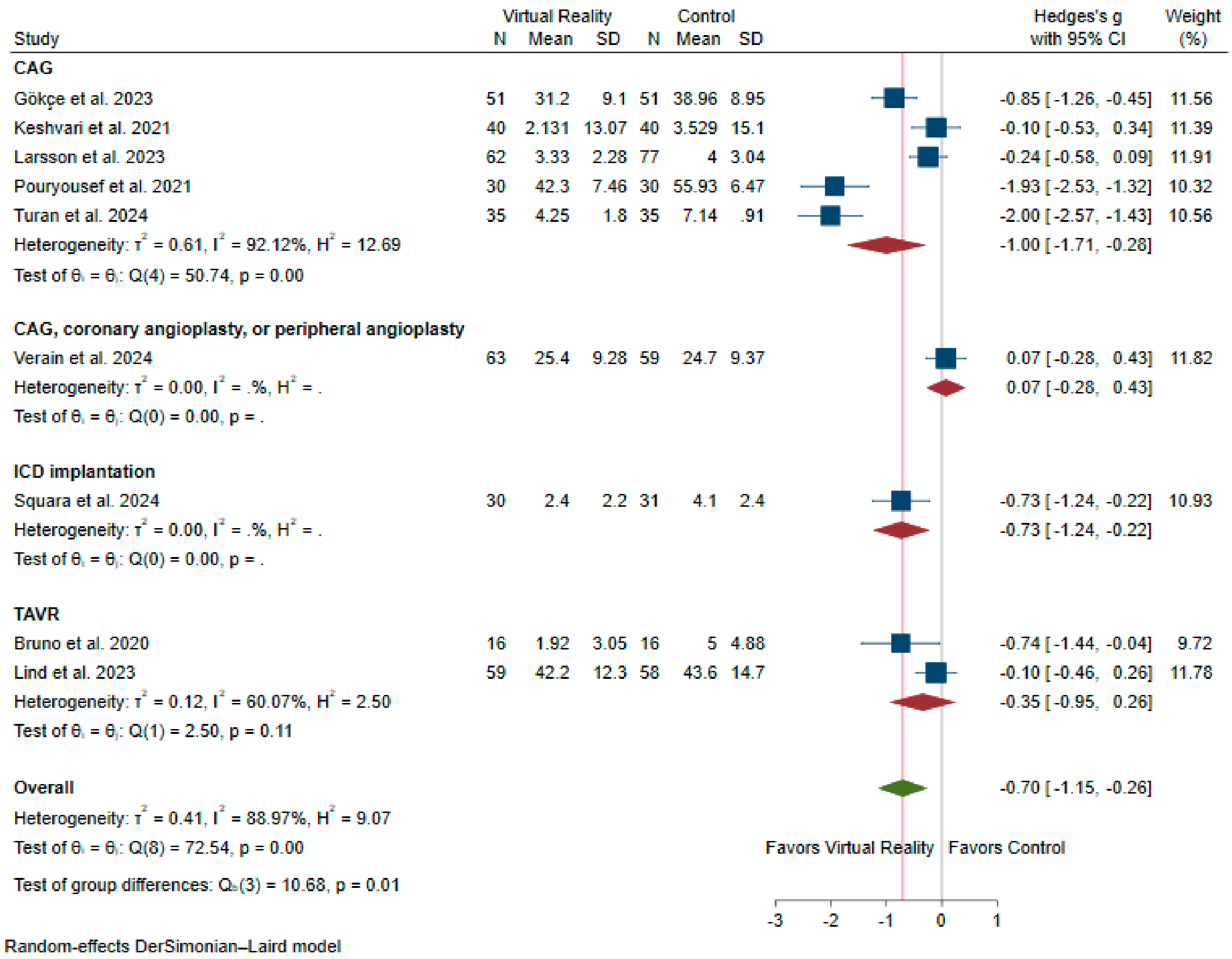

Figure S6: Subgroup analysis based on procedure type in peri-procedural anxiety.

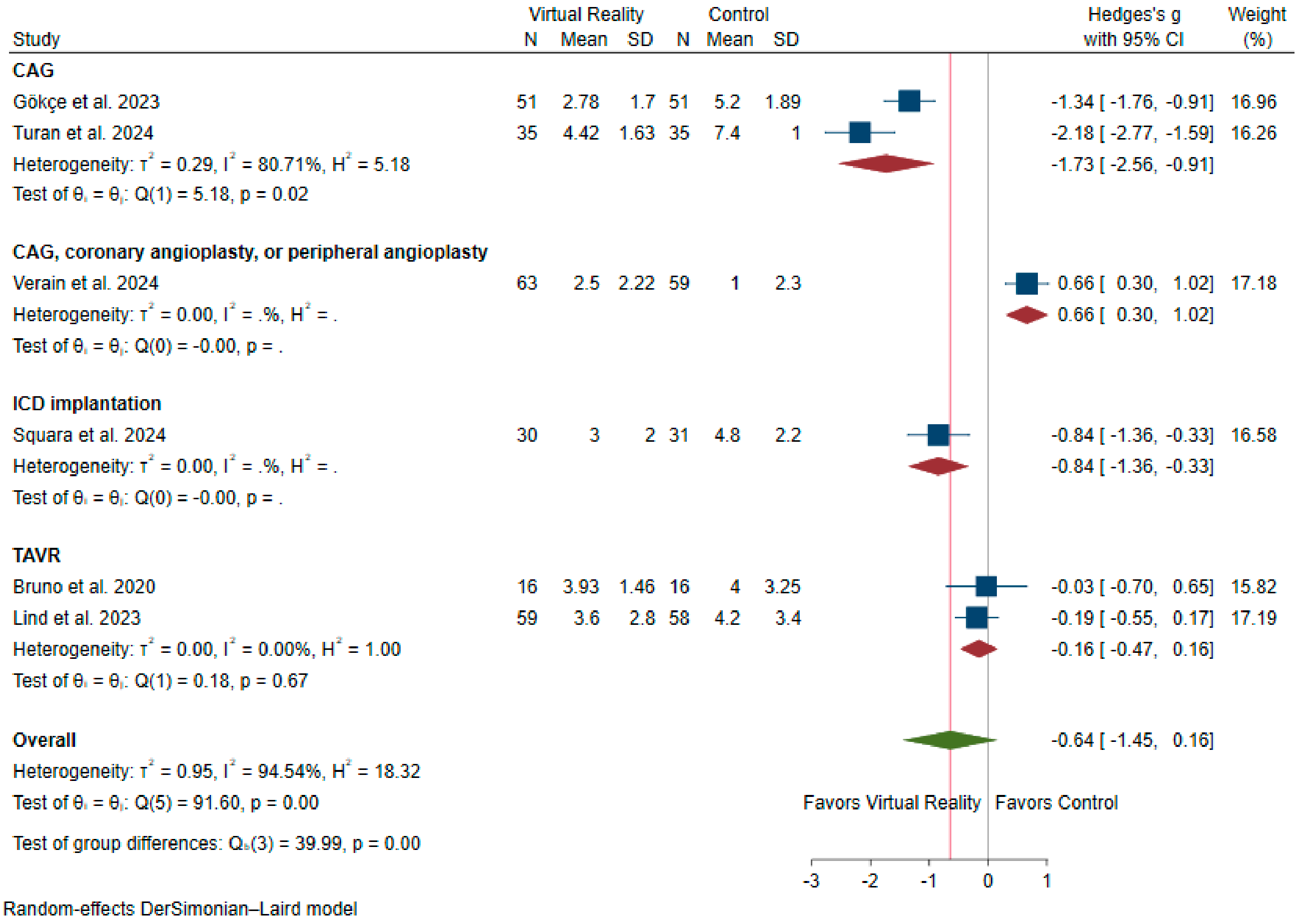

Figure S7: Subgroup analysis based on procedure type in peri-procedural pain.

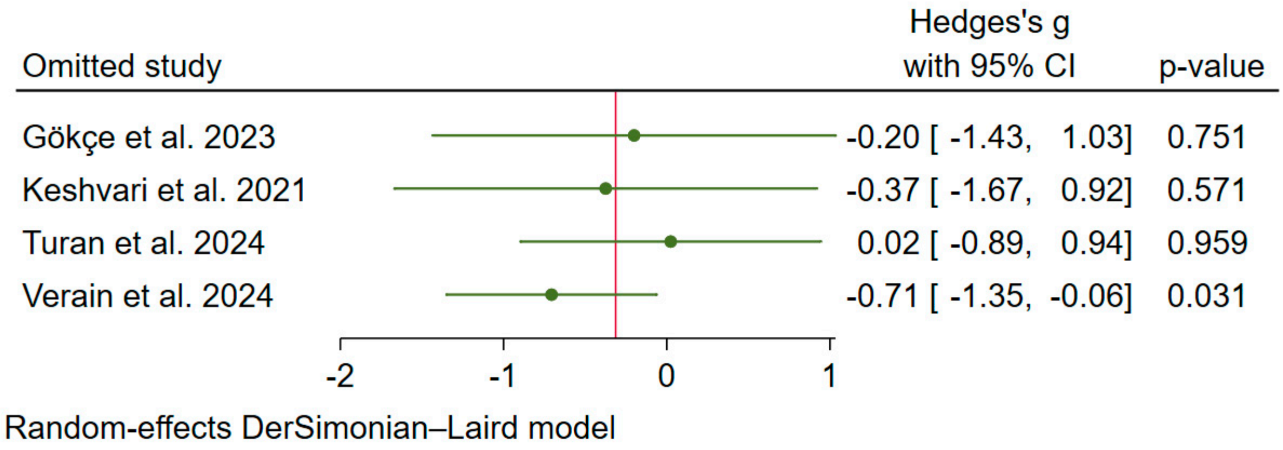

Figure S8: Leave-one-out sensitivity analysis of systolic blood pressure.

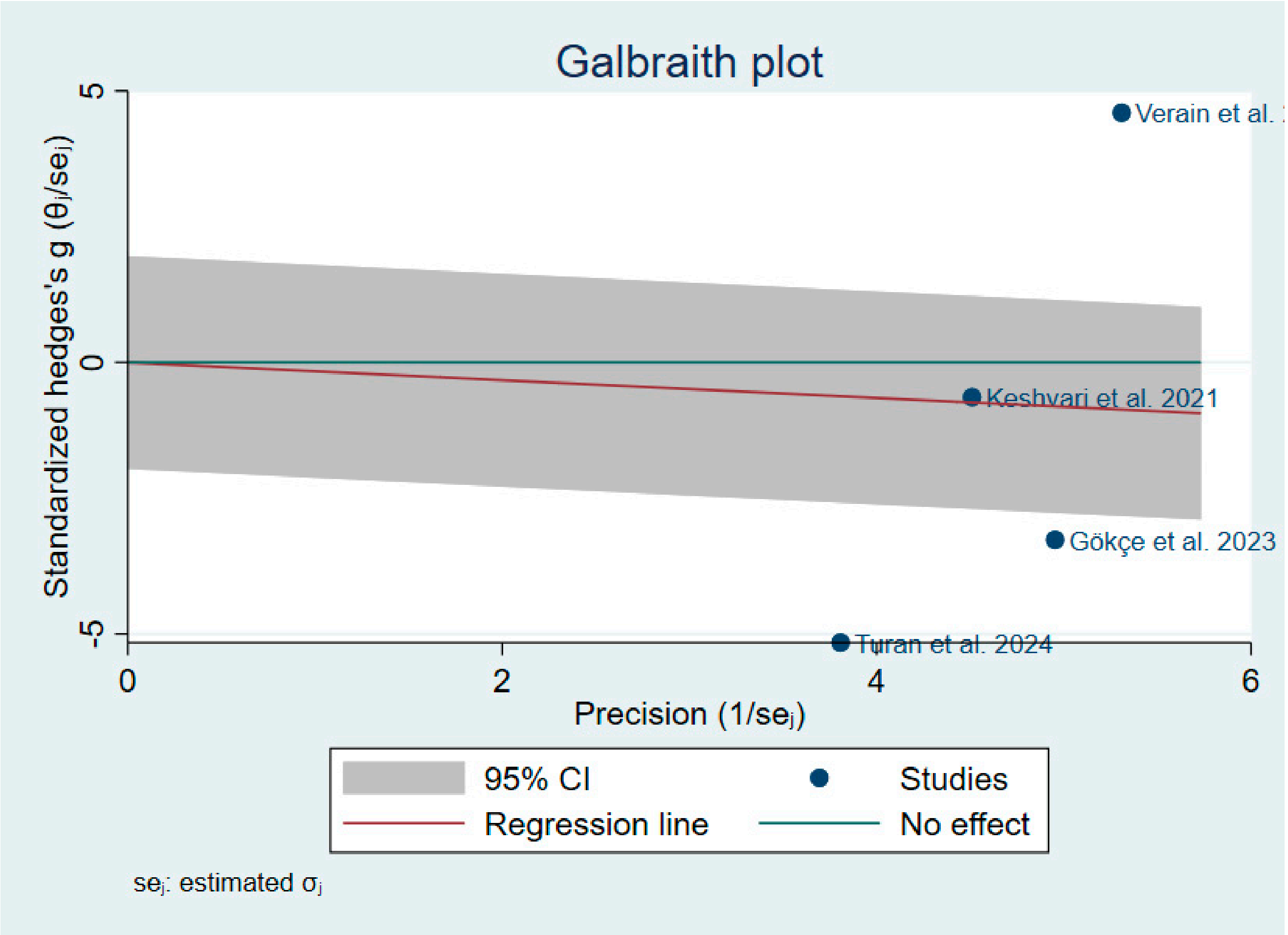

Figure S9: Galbraith plot of systolic blood pressure.

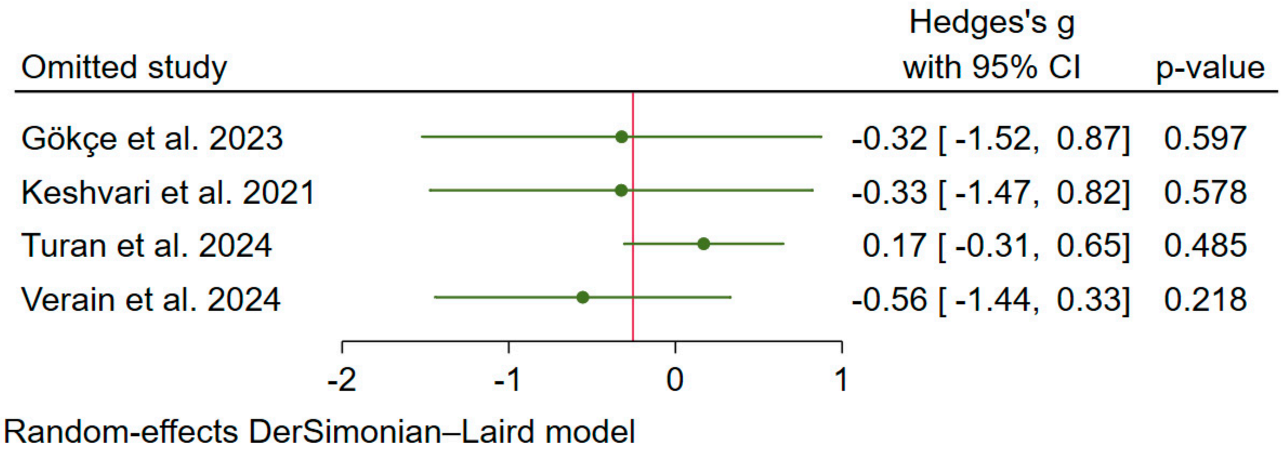

Figure S10: Leave-one-out sensitivity analysis of diastolic blood pressure.

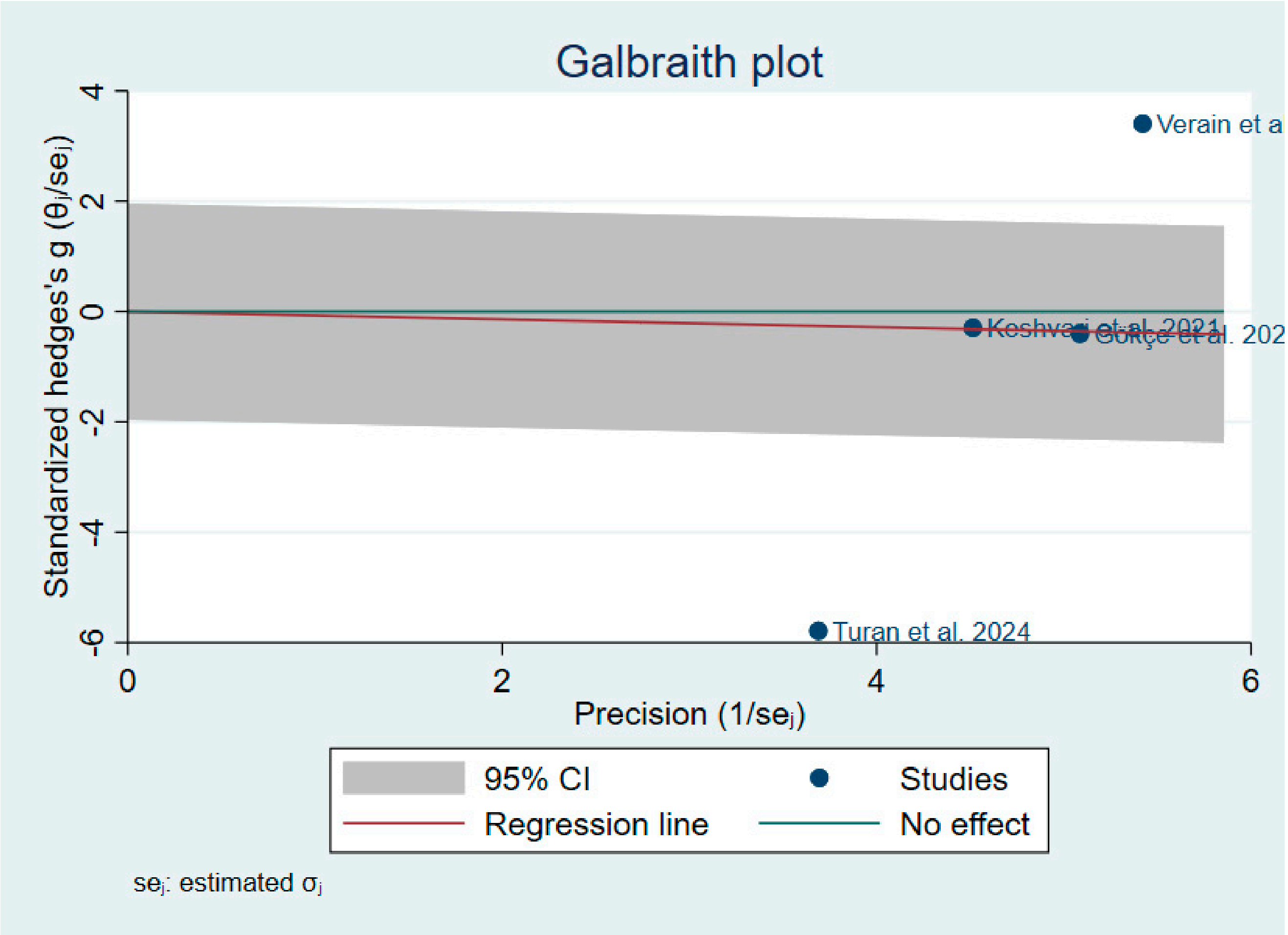

Figure S11: Galbraith plot of diastolic blood pressure.

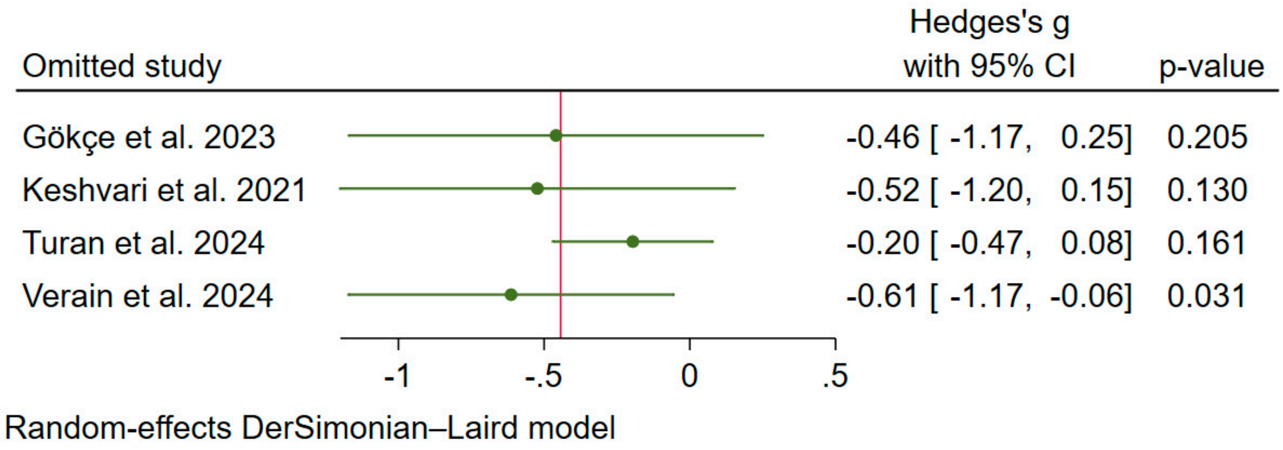

Figure S12: Leave-one-out sensitivity analysis of heart rate.

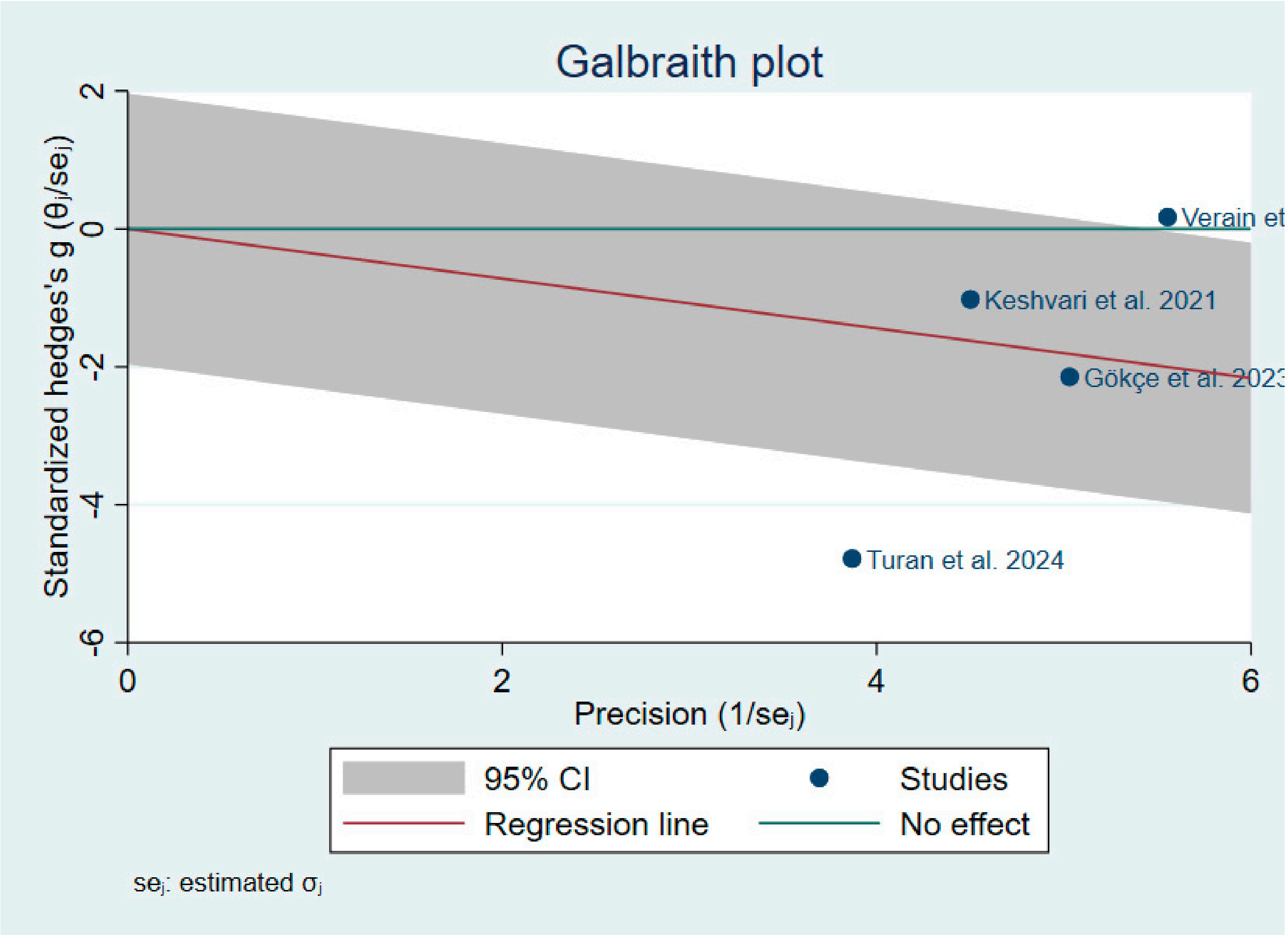

Figure S13: Galbraith plot of heart rate.

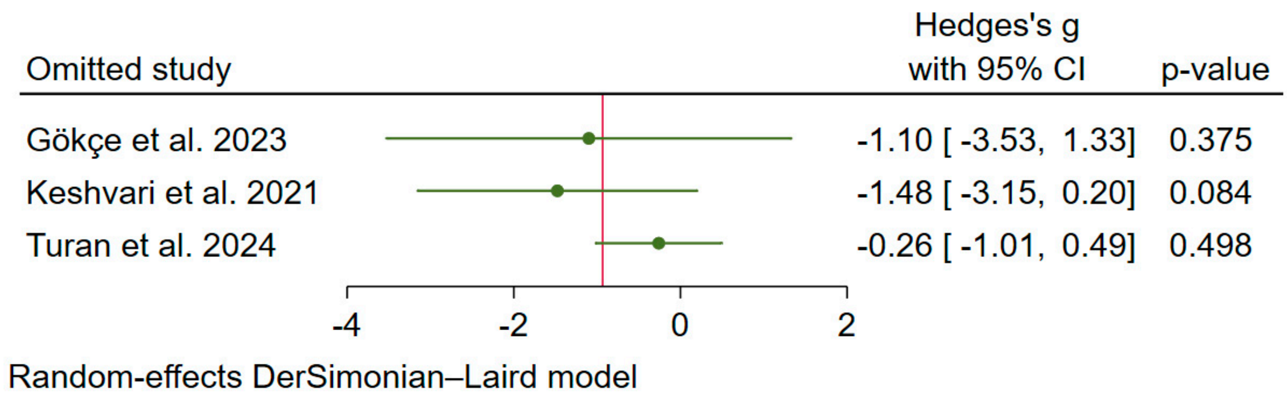

Figure S14: Leave-one-out sensitivity analysis of respiratory rate.

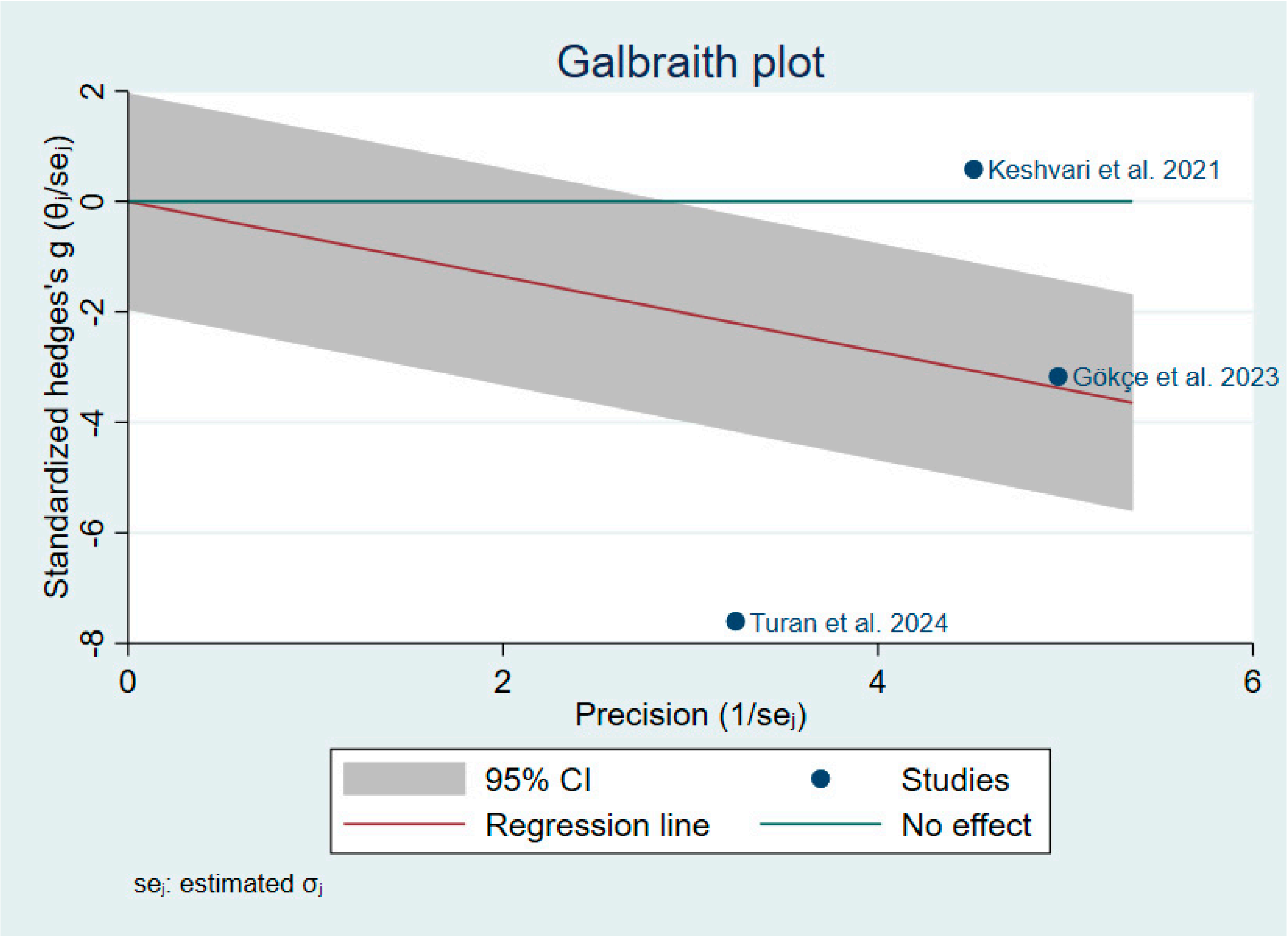

Figure S15: Galbraith plot of respiratory rate.

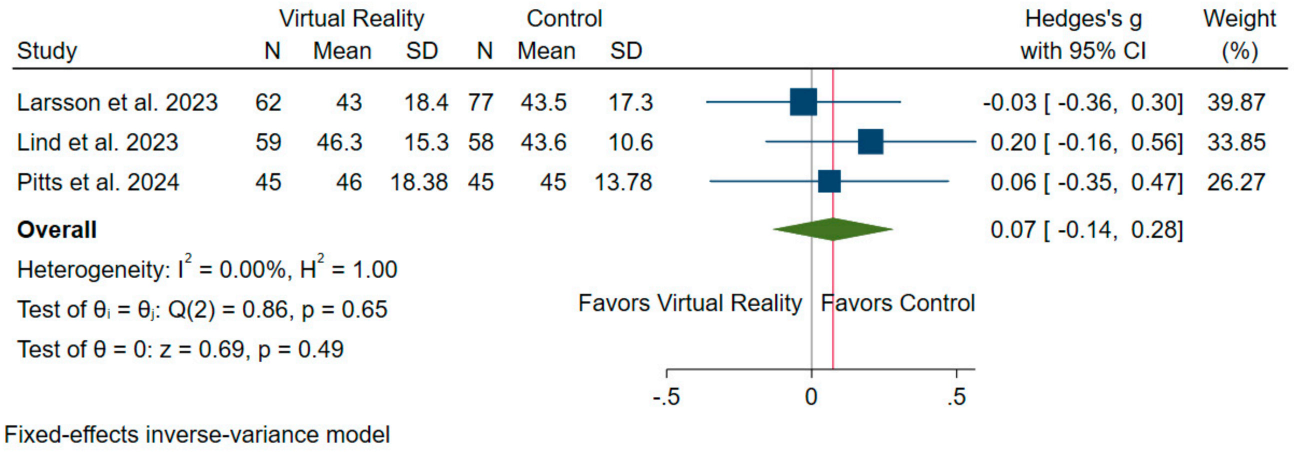

Figure S16: Forest plot of procedure duration.

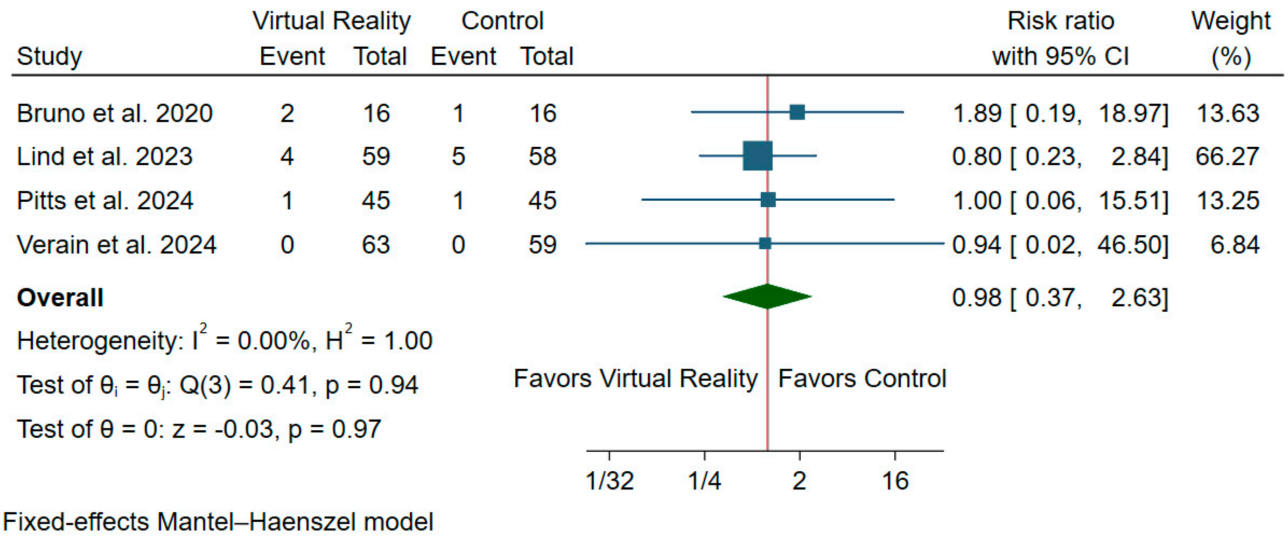

Figure S17: Forest plot of delirium.
